# Microbiomic Analysis on Low Abundant Respiratory Biomass Samples; Improved Recovery of Microbial DNA From Bronchoalveolar Lavage Fluid

**DOI:** 10.3389/fmicb.2020.572504

**Published:** 2020-10-06

**Authors:** Montserrat Saladié, Jose Antonio Caparrós-Martín, Patricia Agudelo-Romero, Peter A. B. Wark, Stephen M. Stick, Fergal O’Gara

**Affiliations:** ^1^Human Microbiome Programme, School of Pharmacy and Biomedical Sciences, Curtin University, Perth, WA, Australia; ^2^Curtin Health Innovation Research Institute, Curtin University, Perth, WA, Australia; ^3^Telethon Kids Institute, Perth, WA, Australia; ^4^ARC Centre for Plant Energy Biology, Faculty of Science, School of Molecular Sciences, The University of Western Australia, Perth, WA, Australia; ^5^Wal-yan Respiratory Research Centre, Telethon Kids Institute, Perth, WA, Australia; ^6^Centre of Excellence in Severe Asthma and Priority Research, Centre for Healthy Lungs, Faculty of Health, University of Newcastle, Newcastle, NSW, Australia; ^7^Department of Respiratory and Sleep Medicine, John Hunter Hospital, Newcastle, NSW, Australia; ^8^Department of Respiratory and Sleep Medicine, Perth Children’s Hospital, Perth, WA, Australia; ^9^BIOMERIT Research Centre, School of Microbiology, University College Cork, Cork, Ireland

**Keywords:** respiratory microbiome, polyethylene glycol, DNA extraction, bronchoalveolar lavage fluid, cystic fibrosis, chronic obstructive pulmonary disease

## Abstract

In recent years the study of the commensal microbiota is driving a remarkable paradigm shift in our understanding of human physiology. However, intrinsic technical difficulties associated with investigating the Microbiomics of some body niches are hampering the development of new knowledge. This is particularly the case when investigating the functional role played by the human microbiota in modulating the physiology of key organ systems. A major hurdle in investigating specific Microbiome communities is linked to low bacterial density and susceptibility to bias caused by environmental contamination. To prevent such inaccuracies due to background processing noise, harmonized tools for Microbiomic and bioinformatics practices have been recommended globally. The fact that the impact of this undesirable variability is negatively correlated with the DNA concentration in the sample highlights the necessity to improve existing DNA isolation protocols. In this report, we developed and tested a protocol to more efficiently recover bacterial DNA from low volumes of bronchoalveolar lavage fluid obtained from infants and adults. We have compared the efficiency of the described method with that of a commercially available kit for microbiome analysis in body fluids. We show that this new methodological approach performs better in terms of extraction efficiency. As opposed to commercial kits, the DNA extracts obtained with this new protocol were clearly distinguishable from the negative extraction controls in terms of 16S copy number and Microbiome community profiles. Altogether, we described a cost-efficient protocol that can facilitate microbiome research in low-biomass human niches.

## Introduction

In recent years, the study of microbial communities colonizing the human body surfaces have revolutionized many fundamental aspects in the areas of medicine and human biology. Now, these communities of microorganisms are considered critical regulators of the normal physiology, and important indicators of pathological processes linked to human disease (Cho and Blaser, 2012; Knight et al., 2017). Exciting developments have highlighted the importance of the homeostatic interaction between the host and its microbiota in health and well-being. The potential role that disturbing these coordinated interactions could play in many human conditions is now center stage. Furthermore, the promise that complementary therapies targeting the reestablishment of balanced communication between host and microbiota could cure disease is gaining traction globally (Cho and Blaser, 2012; Knight et al., 2017; van de Guchte et al., 2018).

Since the preconceived assumption of being a sterile environment, the lower airways and specifically the lungs, have been largely neglected in microbiome research. This fact has delayed the realization that chronic respiratory conditions are greatly influenced not only by the local microbiota of the airways, but also by communities inhabiting niches farther away such as those from the gut (Marsland et al., 2015; Dickson et al., 2016b; Budden et al., 2017, 2019; Faner et al., 2017). Additional pitfalls in the systematic investigation of the respiratory microbiota rely on the low microbial biomass characteristic of this environment, and the susceptibility of microbial contamination from the oropharynx during sampling of the lower respiratory tract. Thus, although the existence of a truly microbial ecosystem in the lungs is still seen with skepticism, rather than settled residents, it is thought that the lung microbiota may actually represent a transient population (Dickson et al., 2015).

With the exception of infective processes involving high microbial burden, the bacterial biomass present in biological specimens from the lungs (usually bronchoalveolar fluid or BALF) is low (Dickson et al., 2016a). This linked to the high sensitivity of the current genomic technologies, make BALF specimens highly susceptible to confounding issues due to contaminant DNA (Salter et al., 2014). The disturbing possibility that many published studies could be dominated, or at least influenced by environmental contaminants, has stimulated thinking onto strategies and best practices to avoid and mitigate the pernicious effect of artificial communities (Eisenhofer et al., 2019). A closely related problem is that most of the taxa widely recognized as typical environmental contaminants, are also truly representatives of the lower respiratory microbiota (Morris et al., 2013; Salter et al., 2014). These issues highlight a major hindrance to get a robust foundation in our understanding of the role played by the microbiota in promoting a healthy respiratory system, especially those processes occurring early in life. Importantly, susceptibility to variation introduced by contaminants is concentration-dependent (Salter et al., 2014). Thus, one strategy to minimize the effect of environmental DNA contamination is developing efficient protocols to improve DNA recovery from these low-biomass specimens.

Different DNA extraction techniques have been used to profile microbial communities from BALF samples; commercial silica column-based kits (Charlson et al., 2011; Willner et al., 2012; Han et al., 2014; Renwick et al., 2014; May et al., 2015; Dickson et al., 2016b; Laguna et al., 2016; Marsh et al., 2016; Wen et al., 2016; Zemanick et al., 2017; Ahmed et al., 2018; Pattaroni et al., 2018; Kyo et al., 2019; Schneeberger et al., 2019; Tong et al., 2019; Wang et al., 2019), magnetic beads-based procedures (Erb-Downward et al., 2011; Pragman et al., 2012; Bernasconi et al., 2016; Frayman et al., 2017; Kloepfer et al., 2018; Esther et al., 2019; Gomes et al., 2019), homemade protocols (Borewicz et al., 2013; Jorth et al., 2019), salt-extraction approaches or the use of cationic detergents in combination with the classical phenol-chloroform extraction technique (Willner et al., 2012). However, not many studies have compared the efficiency of these protocols, particularly using samples from young children (Willner et al., 2012). BALF specimens from very young individuals constitute a rare opportunity of evaluating early host-microbiota coordinated interactions (Pattaroni et al., 2018). In many cases these unique samples are volume-limited, requiring an efficient DNA extraction protocol to minimize the susceptibility of introducing unwanted variability (Salter et al., 2014).

In this report, we present an efficient extraction protocol to recover bacterial DNA from BALF supernatants sampled from cystic fibrosis (CF) infants and chronic obstructive pulmonary disease (COPD) adults. Our approach combines an optimized mixture of hydrolytic enzymes to improve the digestion of cell-wall components (Tighe et al., 2017), with the polyethylene glycol (PEG)-induced *ψ* condensation of DNA in the presence of NaCl (Cheng et al., 2015). We also compare the performance of this new method with that of a commercial kit optimized for extracting DNA from swabs and body fluids (QIAamp Microbiome Kit, Qiagen, 51704), using a customized TaqMan^®^ qPCR assay. Demonstrating the adequacy of the DNA isolated with this new protocol for downstream high throughput applications, we submitted the DNA extracts to *16S rRNA* gene amplicon sequencing. In deep contrast with the column-based DNA extracts, the communities associated with the PEG protocol were clearly distinguishable from the corresponding extraction negative controls, suggesting that the choice of the DNA extraction protocol has dramatic effects in the study of communities with low microbial density.

## Materials and Methods

### Patient Samples

Two different cohorts of BALF specimens were used in this study. The first cohort consisted of 5 BALF samples from adults (age range 45–82 years; median 55, interquartile range (IQR) 48–76). Four specimens were from patients diagnosed with chronic obstructive pulmonary disease (COPD, GOLD stage 1–4). The fifth sample was from a healthy smoker individual. Subjects in this cohort were without acute infection at the time of BALF sampling and were asked to confirm that they did not have symptoms of infection or antibiotic use in the previous 6 weeks. The protocol used for collecting the BALF specimens in the COPD cohort was as previously described (Lokwani et al., 2019).

The second cohort was a subset of 6 infants diagnosed with cystic fibrosis (CF) enrolled in AREST-CF surveillance program. These patients were asymptomatic at the time of BALF acquisition. These babies ranged in age from 0.28 to 0.91 years old (median age 0.63; IQR 0.36–0.79) at the time of the bronchial wash collection and were sampled during the follow-up annual visit as previously described (Mott et al., 2012; Caparros-Martin et al., 2020).

### Pre-processing of the BALF Aliquots

All the steps described in this paper were performed under sterility conditions in a laminar flow cabinet located in a pre-PCR room. Each BALF aliquot (1 mL) was centrifuged at 20,000 × g for 30 min at 4°C. The supernatant was discarded and the pellets were carefully resuspended in 100 μL of HyClone Phosphate Buffered Saline solution (PBS) pH 7.5 without EDTA (GE Healthcare, Life Sciences, United Kingdom) by pipetting up and down using filter barrier tips.

### DNA Extraction Procedures Using Commercially Available Kits

We used a column-based commercial kit from Qiagen; the QIAamp DNA microbiome kit (Qiagen, 51704). The purification principle for this protocol is based on the ionic interactions between the DNA and the silica columns. Extraction was strictly performed following the manufacturer instructions except for the elution volume that was set to 25 μL, and a bead-beating pre-treatment that was added to improve cell lysis. For this purpose, resuspended cell pellets were bead-beaten with 0.1 g of zirconia/silica beads (0.1 mm diameter. Daintree Scientific) in a cell disrupter (Mini beadbeater-16^TM^, Biospec) using 4 pulses of 1 min each. We did also test the performance of host depletion in increasing the recovering efficiency of bacterial DNA from BALF samples. Removal of host DNA was performed following the protocols and reagents provided within the QiaAmp microbiome kit.

### Polyethylene Glycol-Based DNA Extraction Protocol

All the reagents used in this protocol are sterile grade products purchased from either SIGMA or Bioworld. Any solution described in this protocol (e.g., enzyme solutions) was prepared in sterile conditions using these commercial reagents. Resuspended pellets were incubated for 4 h at 35°C with 20 μL of MetaPolyzyme solution (10 mg/mL in Hyclone PBS pH 7.5 without EDTA) (MAC4L, Sigma-Aldrich) (Tighe et al., 2017). Then, a second incubation with 10 μL of proteinase K (10 ng/mL, Sigma-Aldrich) for 1 h at 56°C was performed. After the second incubation, samples were stored at 80°C overnight. Twelve hours later, tubes were thaw on ice and cells crushed using a bead-beater as described above. Tubes were then briefly centrifuged (5,000 × g for 1 min at 4°C). Supernatants were carefully removed and placed in a new tube. The pellets were resuspended in 100 μL of PBS solution and an additional cycle of bead beating-centrifugation performed. Combined supernatants were extracted with one volume of 24:1 chloroform:isoamyl alcohol solution (25666. Sigma-Aldrich). After vigorous vortexing, tubes were centrifuged (20,000 × g at 4°C for 10 min). The nucleic acid-containing aqueous phase was carefully removed and placed in a new tube. A back-extraction was performed by adding 50 μL of PBS to the organic (chloroform:isoamyl alcohol) phase. After vortexing and centrifugation, the second and first aqueous phases of the same sample were combined. Nucleic acids were precipitated with 1.5 volumes of sterile 30% polyethylene glycol 8,000 solution in 1.6 M NaCl pH 6.7 (41620040-1, Bioworld) for 2 h on ice. After this incubation, samples were centrifuged (20,000 × g at 4°C for 30 min). The supernatant was discarded and the pellet washed with 800 μL of a 70% ethanol solution prepared with 200 proof ethyl alcohol (SIGMA, E7023), and sterile molecular biology-grade water (W4502, Sigma-Aldrich). The pellet was then air dried and resuspended with 25 μL of filtered (0.1 μm) sterile molecular biology-grade water (W4502, Sigma-Aldrich).

### *16S rRNA* Gene PCR Amplification

Concentration of DNA was evaluated using a NanoDrop spectrophotometer. PCR amplification was performed as follows. One microliter of DNA was used to amplify a fragment of the *16S rRNA* gene using the primers 63F (5′-*CAGGCCTAACACATGCAAGTC*-3′) and 1387R (5′-*GGGCGGWGTGTACAAGGC*-3′) at a final concentration of 0.4 pmol μL^–1^ (Marchesi et al., 1998). For amplification we used MyTaq DNA polymerase (Bioline, London, United Kingdom) and nuclease-free water was added to a final volume of 25 μL. PCR conditions were as follow: initial denaturation at 95°C for 3 min, then 30 cycles (95°C for 30 s, 60°C for 30 s and 72°C for 60 s), following a final extension step at 72°C for 5 min. Negative controls, containing all the components except DNA templates, were run in parallel. We also included technical controls consisting of a DNA extraction negative control. PCR products were subjected to 1% (w/v) agarose gel electrophoresis to confirm the amplification of a single product of the expected size.

### *16S rRNA* Gene-Based BALF-Associated Microbiota Profiling

For 16S rRNA amplicon – based microbial profiling, DNA extracts were used to generate amplicons using a MetaVx^TM^ Library Preparation kit (GENEWIZ, Inc., South Plainfield, NJ, United States) at Genewiz. The V3-V4 hypervariable region of the *16S rRNA* gene was targeted and amplified using forward and reverse primers containing the sequences 5′-*CCTACGGRRBGCASCAGKVRVGAAT* and 5′-*GGACTACNVGGGTWTCTAATCC*, respectively. The DNA amplicon libraries were validated using Agilent 2100 Bioanalyzer (Agilent Technologies, Palo Alto, CA, United States) and quantified using Qubit 2.0 Fluorometer. Then, the libraries were multiplexed and sequenced on a MiSeq^®^ instrument (Illumina, San Diego, CA, United States) using a 2 × 300 paired-end configuration accordingly to manufacturer’s recommendations.

Pre-processing of the sequencing data was carried out using custom shell scripts. Briefly, quality of the raw data was evaluated using FastQC and MultiQC (Ewels et al., 2016). Based on the generated quality reports, raw data was trimmed and cropped using Trimmomatic to remove low quality reads and any remaining Illumina adapter (Bolger et al., 2014). Paired-end reads were then joint using BBMerge (Bushnell et al., 2017). After pre-processing the sequencing data, we obtained more than 6 million paired end reads (length 250 bp) of high quality sequences (average quality Q30). For Operational Taxonomic Unit (OTU) assignment, reads were clustered at a 98% threshold using the SILVAngs analytical pipeline as previously described (Quast et al., 2013). For taxonomic classification a local BLAST search was done against the non-redundant version of the SILVA SSU Reference (release 132)^1^ using blastn (Camacho et al., 2009). OTUs representing less than 0.01% across all samples were considered low-count taxa and were removed. This approach has previously been used to avoid inflation of diversity estimates due to OTUs representing potential PCR-related artifacts (Le Cao et al., 2016). After pre-processing the taxonomic table, we took advance of the functions implemented in the R package *decontam* to identify and to eliminate OTUs likely originated from contamination using the negative extraction controls (Davis et al., 2018). These putative contaminants are provided in Supplementary Table 5 and were fully removed from the corresponding biological specimens. After removing background contaminants, OTU profiles were transformed to relative proportions. Compositional data was then subjected to centered-log ratio transformation to map data into Cartesian space before performing multivariate analysis (Le Cao et al., 2016; Rohart et al., 2017). Principal component analysis was calculated using the *pca* function as implemented in the R package mixOmics, which uses singular value decomposition for spectral decomposition (Rohart et al., 2017). OTU profiles were quantified by multiplying the compositional taxonomic table by the total bacterial biomass value obtained through qPCR as previously described (Jorth et al., 2019). Analysis of similarities (ANOSIM) was performed on this quantified OTU table using a Bray-Curtis distance metric.

We used R (version 3.6.1) for data analysis using both built-in and custom-made functions (R Core Team, 2017). When using R packages, functions were implemented following the recommendation of the authors in the package vignettes. Lung microbiota composition was evaluated using the MixMC framework implemented in the R package *mixOmics* (Le Cao et al., 2016; Rohart et al., 2017). Other packages used in this study were *vegan* for Procrustes and ANOSIM analyses (Oksanen et al., 2016), *decontam* (Davis et al., 2018), and *PairedData* (Champely, 2018) and *ggplot2* (Wickham, 2016) for visualization.

### Absolute Quantification of Bacterial Biomass Using Quantitative PCR

For quantitation of bacterial DNA we used a Custom TaqMan^®^ Gene Expression Assay Design based on a previously reported universal probe for Bacteria (Nadkarni et al., 2002). *Stenotrophomonas maltophilia*-specific qPCR assay was re-designed from that published by Fraser et al. (2019), to fit with the TaqMan assay conditions recommended by Applied Biosystems. Both TaqMan^®^ probes were labeled with a fluorescein dye (FAM) on the 5′ end, and a minor groove binder quencher (MGB) on the 3′. Details about the experimental conditions are provided in Supplementary Table 8 following the MIQE guidelines (Bustin et al., 2009). The PCR reaction mix consisted of 10 μL of TaqMan^®^ Fast Advanced Master Mix (2X), 1 μL of TaqMan^®^ Assay (20X), 7 μL of nuclease-free water and 2 μL of DNA extract, per reaction. Assays were performed in TaqMan^®^ Fast 96-well plates and were run on a ViiA^®^ 7 Real-Time PCR System instrument under recommended conditions (50° for 2 min; AmpliTaq^TM^ Fast DNA Polymerase activation: 95° for 5 min and then PCR reaction: 40 cycles of 95°, 1 s and 60°, 20 s). Absolute quantification was achieved using a standard curve approach. A serial dilution of microbial DNA standard from *Pseudomonas aeruginosa* (SIGMA, MBD0014) or from *Stenotrophomonas maltophilia* (Minerva biolabs, DSM50170), was used for the universal probe and for the *Stenotrophomonas maltophilia-*specific TaqMan^®^ assays, respectively.

### Ethics, Consent and Permissions

Ethical approval related to the CF patients was granted to the AREST CF program by the Princess Margaret Hospital for Children, Perth ethics committee (Ref. 1762/EPP. Date of approval December 10th, 2009). The ethical aspects related to the COPD cohort was approved by Hunter New England LHD Ethics committee [Mechanisms of Inflammatory airways disease (05/08/10/3.09)]. Informed consent for publication and participation in this study was obtained from the patients or guardians.

### Data Availability

All the data required to reproduce the results of this study are within the manuscript and its supporting information files. The code used in the analysis in this manuscript can be made available for appropriate scientific purposes upon request to the authors. Demultiplexed FASTQ files are deposited in the Sequencing Read Archive under the Bioproject PRJNA636842.

## Results

### Microbiomic Related Rationale for Developing a New DNA Extraction Protocol

Several methods have been described in the literature to isolate bacterial DNA from BALF (Supplementary Table 1; Charlson et al., 2011; Erb-Downward et al., 2011; Pragman et al., 2012; Willner et al., 2012; Borewicz et al., 2013; Han et al., 2014; Renwick et al., 2014; May et al., 2015; Bernasconi et al., 2016; Dickson et al., 2016a,b; Laguna et al., 2016; Marsh et al., 2016; Wen et al., 2016; Frayman et al., 2017; Zemanick et al., 2017; Ahmed et al., 2018; Kloepfer et al., 2018; Pattaroni et al., 2018; Esther et al., 2019; Gomes et al., 2019; Jorth et al., 2019; Kyo et al., 2019; Schneeberger et al., 2019; Tong et al., 2019; Wang et al., 2019). In some instances, successful recovery of DNA has been associated with the utilization of large volumes of starting material. However, in some circumstances and particularly in the case of infants this is not always possible to achieve. Work in our laboratory using both silica column- or magnetic bead-based procedures to isolate DNA from low volumes (∼1 mL) of bronchial washing supernatants, have exhibited non-optimal bacterial DNA extraction yields unless the sample was associated with an evident bronchial infection. This situation is not particularly satisfactory when the amount of biological material that can be retrieved through bronchial washing is limited, as normally applies to newborn and infant cohorts. Polyethylene-glycol (PEG)-based methods have successfully been used to improve the efficiency of recovering bacteriophages, exosomes or edible nanoparticles (Castro-Mejia et al., 2015; Deregibus et al., 2016; Garcia-Romero et al., 2019; Kalarikkal et al., 2020). These methods have also been shown to improve the yield and purity of microbial DNA recovered from different low-biomass ecological niches (Arbeli and Fuentes, 2007). Mechanistically, PEG induces the condensation of DNA molecules in the presence of salts, a process also called *ψ* condensation (Cheng et al., 2015). In view of these positive results, we reasoned that combining an improved cell lysis step and the PEG-induced DNA precipitation would result in a significant improvement in the recovery of bacterial DNA from reduced volumes of low-biomass BALF. Details of this protocol are provided in the material and methods section and Supplementary Figure 1.

### The PEG-Based Protocol Shows Better Recovery of Bacterial DNA Compared to a Column-Based Kit

We evaluated the efficiency of three different extraction protocols in isolating microbial DNA from reduced volumes of low-biomass BALF samples. Two or three equal volume aliquots (1 mL) per patient were processed using three different protocols: (a) the PEG protocol described in this report and (b) the QIAamp microbiome kit (Qiagen, 51704). For those specimens for which 3 mL of BALF were available, matched aliquots of the extracted DNA were either untreated or (c) subjected to the optional treatment to deplete host DNA. Clinical details of the study subjects are provided in Supplementary Table 2.

**FIGURE 1:**
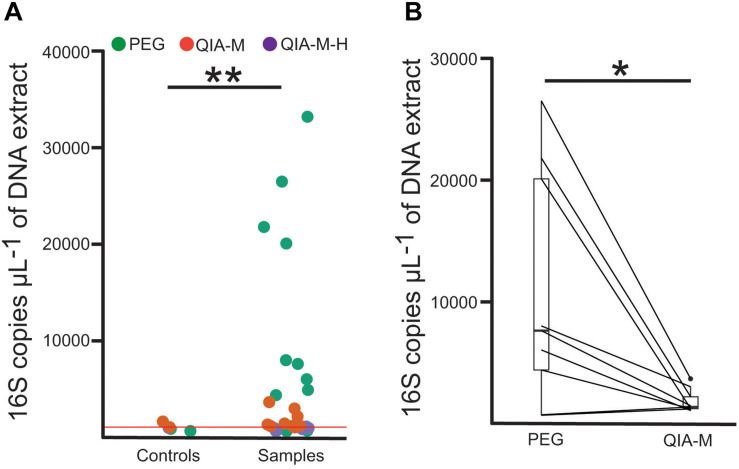
The PEG-based method exhibits the best bacterial DNA recovery efficiency. **(A)** 16S copy number assayed through qPCR in samples and negative extraction controls. Each dot represents an independent extract and are colored accordingly to the extraction method used. The red horizontal line represents the average number of 16S copies observed in the negative extraction controls. PEG, polyethyelene glycol; QIA-M, QIAamp microbiome kit; QIA-M-H, QIAamp microbiome kit with DNA host depletion. **(B)** Barplots represent the amount of bacterial DNA recovered in matched aliquots (connected using straight lines) from the same BALF specimen using the PEG-based (PEG) or the column-based (QIA-M) kit. Unpaired **(A)** or paired **(B)** Welch *t*-test was used for statistical inference. *P < 0.05; **P < 0.01.

Levels of bacterial DNA were evaluated employing a quantitative PCR (qPCR) custom assay. As indicated from the results of the qPCR experiments, the overall performance of the PEG-based protocol was superior to the column-based kit (Figure 1 and Supplementary Table 3). Success in the isolation of bacterial nucleic acids was further confirmed by selectively amplifying a specific fragment of the *16S rRNA* gene (Supplementary Figure 2). In four of the bronchial wash specimens from CF infants, the number of 16S copies obtained with the PEG protocol was higher than the background present in the negative extraction control (Supplementary Table 3). Interestingly, the amount of bacterial DNA retrieved from these samples using the QIAamp microbiome kit was indistinguishable from the corresponding extraction control. This was independent of whether a host depletion step was included. This data suggests that the differences in DNA recovery are likely to be due to either a suboptimal interaction between the DNA and silica surfaces, or a lysis-related extraction bias. On the other hand, similar results were obtained for the BALF samples from the adult cohort, with the PEG protocol producing DNA extracts with higher number of 16S copies than the commercial kit (Figure 1 and Supplementary Table 3). Interestingly, we observed that the number of 16S copies present in the negative extraction controls using the PEG control trended to be lower than the background noise obtained with the column-based kit [mean(*SD*), PEG: 761.12(158.02); QIAamp microbiome kit: 1218.1(362.3); Welch *t*-test 0.19] (Supplementary Table 3).

### DNA Extracted With the PEG-Based Protocol Is Suitable for Downstream Sequencing and Microbiome Analysis

Commercial kits ensure high quality DNA extracts, which are virtually PCR inhibitors-free. Such DNA samples are appropriate for high-throughput downstream applications. Absence of PCR inhibitors in the PEG extracts was confirmed by conventional PCR targeting a 1,344 bp DNA fragment of the *16S rRNA* gene (Supplementary Figure 2). We also tested whether the DNA obtained with the PEG protocol was suitable for 16S rRNA amplicon sequencing. For this approach, we targeted the V3-V4 hypervariable region of the *16S rRNA* gene. Amplicon libraries from 31 samples including 5 negative extraction controls were generated and sequenced at Genewiz (Suzhou, China). We obtained 6,679,284 paired-end reads (single length of 250 bp) with a high base calling accuracy (median Phred-like Q-score of 30, IQR 25–37).

After progressing the specific filtering steps described in the methods section, we obtained an OTU profile representing 231 bacterial taxa. As recommended by harmonized global publication data standards (Eisenhofer et al., 2019), we are reporting the taxa detected in negative extraction controls as well as their absolute abundance (Supplementary Table 4). As expected, negative extraction controls presented significantly lower bacterial biomass than the targeted biological specimens (Figure 1A). We also observed that the number of reads in the blank extraction controls was also reduced (controls: median 16,327, IQR 10,665–21,849; biological specimens: median 33,786, IQR 21,153–45,552. Welch *t*-test *p-*value = 0.002). However, total biomass was not linearly correlated with the number of reads, confirming that this variable is not a reliable surrogate of bacterial density [*F*(1, 29) = 1.889, *p*-value 0.18, multiple *R*^2^ 0.06]. To evaluate the influence of the environmental background associated with each extraction procedure in the taxonomic profiles of the targeted biological specimens, we also made use of the R package *decontam* (Davis et al., 2018). We identified and filtered out putative contaminants by applying the “combined” method implemented in the function is contaminant (Davis et al., 2018; Supplementary Table 5).

**FIGURE 2:**
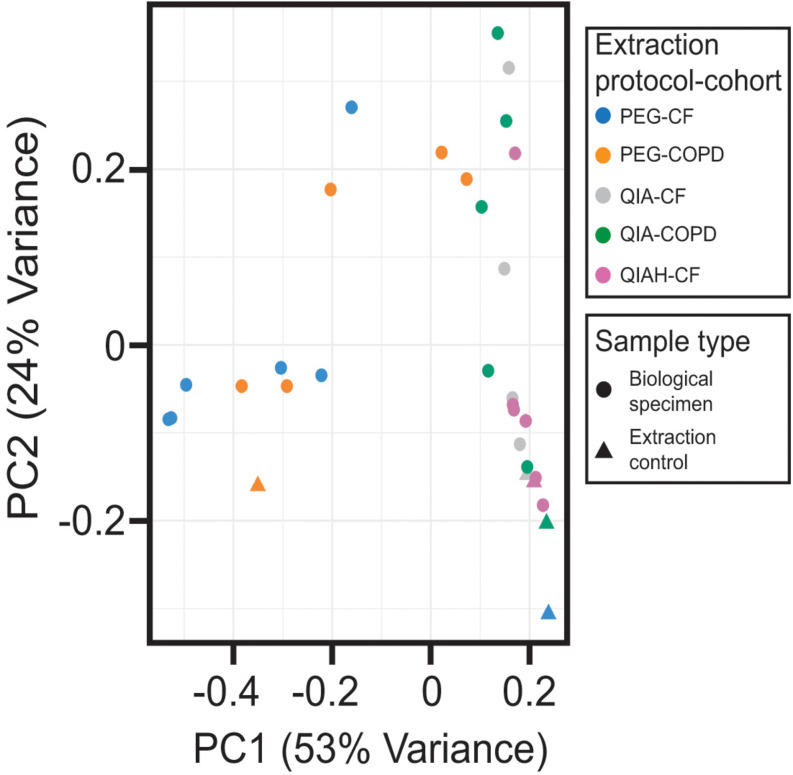
Multivariate analysis of the 16S-based taxonomic profiles. Projection of the two principal components (PC) of the principal component analysis model. Dots and triangles represent biological specimens and negative extraction controls, respectively. Colors represent the different extraction method used. PEG, polyethylene glycol; QIA, QIAamp microbiome kit; QIAH, QIAamp microbiome kit with DNA host depletion; CF, cystic fibrosis; COPD, chronic obstructive pulmonary disease.

We next evaluated the microbial communities present in both the clinical samples and the extraction controls, using a principal component analysis model (Figure 2). Inspection of the first two components of the model revealed that the samples processed with the column-based kit were closely located to their corresponding negative extraction controls. These samples were distributed along the component accounting for the lower proportion of explained variance (component 2), as opposed to the samples extracted with the PEG method (Figure 2). Thus, the microbial communities profiled from the PEG DNA extracts yielding a number of 16S copies higher than the background noise, were clearly distinguishable from the extraction negative controls (Analysis of similarities, ANOSIM. global *R* = 0.7748, *p* < 0.05). These observations suggest that the biological variability captured by the PEG method is higher than that obtained after processing the samples with the commercial kit. We speculated that this could be a consequence of the higher DNA recovery exhibited by this extraction protocol (Figure 1B and Supplementary Table 3). To test this hypothesis, we calculated the Procrustes distance between paired samples and related this to the absolute difference in bacterial biomass between them using a linear model. The outcome of this analysis confirmed that the higher the difference is in concentration the larger the Procrustes distances are between paired samples [*F*(1, 13) = 4.706, *p*-value 0.049, multiple *R*^2^ 0.26].

**FIGURE 3:**
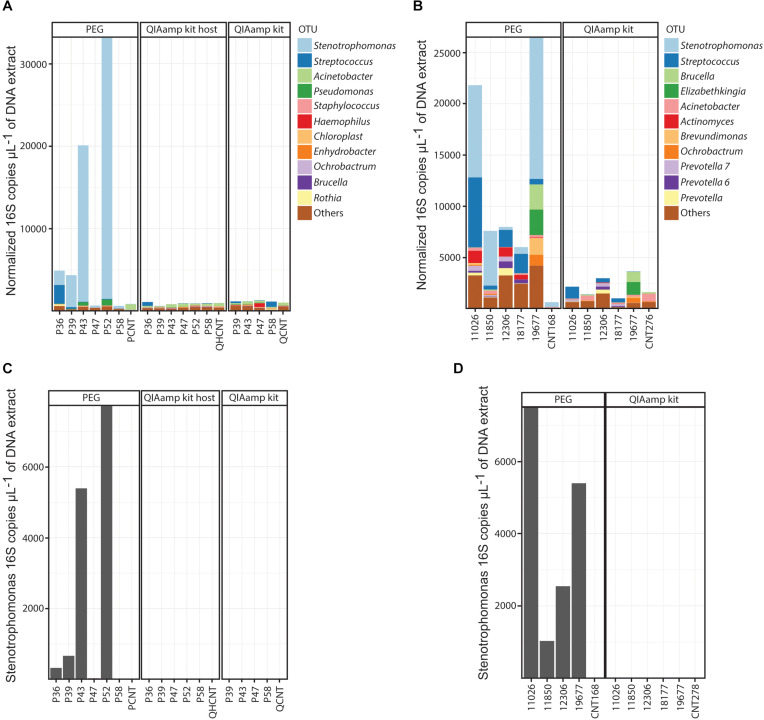
OTU compositional profiles of the BALF-associated microbial communities. **(A,B)** Normalized profiles of the top 11 taxa observed in BALF from CF **(A)** or COPD **(B)** patients. **(C,D)** Normalized levels of *Stenotrophomonas maltophilia* in DNA extracts obtained from CF **(C)** and COPD **(D)** BALF specimens. PEG, polyethylene glycol; QIAamp kit, QIAamp microbiome kit; QIAamp kit host, QIAamp microbiome kit with DNA host depletion.

Finally, we analyzed the composition of the resulting microbial communities present in the BALF specimens (Figures 3A,B). Accordingly with the PEG extractions, bronchial washings from CF infants were less diverse than BALF specimens from COPD adults [Richness. Mean (Standard deviation, *SD*) CF cohort: 42.6(11.4), mean (*SD*) adult cohort: 76.4(7.7). Welch *t*-test *p*-value 0.0002] (Supplementary Figure 3 and Supplementary Table 6). Bronchial wash-associated communities in CF infants were characterized by the presence of oral flora (*Streptococcus* and *Rothia*) and Proteobacteria (*Stenotrophomonas* and *Pseudomonas*) (Figure 3A). On the contrary, microbial communities associated with BALF from COPD adults exhibited a higher richness (Supplementary Figure 3 and Supplementary Table 6). In general the communities from adult individuals were dominated by OTUs assigned to taxa that are typical colonizers of the human oral cavity (Aas et al., 2005). We found interesting that the microbial profiles of COPD patients classified as GOLD1–3 categories (samples 18177, 12306, and 11026) were similar, with an evident increase in bacterial load as disease progresses (GOLD 1–4, samples 18177, 12306, 11026, and 19677) (Supplementary Table 3). Conversely, the BALF-associated microbiota of the patient classified as GOLD4 (19677) was characterized by the presence of opportunistic oral pathogens such as *Brucella* or *Elizabethkingia* (Figure 3B). Further analysis using larger cohorts will address whether this microbial succession in the lungs of COPD patients play a role in disease progression. In the case of samples extracted with the column-based protocol, the OTU profiles were similar to those of the extraction controls (column-based no host depletion method: ANOSIM global *R* = 0, *p*-value = 0.46), except for those samples for which a higher concentration of DNA was retrieved (e.g., sample Q19677 and Q12306, Figure 3B and Supplementary Table 3). In these instances, the resulting communities were similar to those profiled in the PEG-associated DNA extracts (ANOSIM, global *R* = 0, *p*-value = 0.66) (Figure 3B). Importantly, the 16S profiles from the PEG extracts in which the bacterial DNA levels were higher than the controls, were consistent with the reported clinical microbiology findings (Supplementary Table 7).

In the adult cohort of BALF specimens, we also identified *Stenotrophomonas* as part of the resulting communities associated with the PEG method. Because of the high prevalence of this pathogen in our cohort, and the ensuing possibility that it could be a contaminant associated with the PEG method, we proceeded with the quantification of *Stenotrophomonas maltophilia* DNA using qPCR (Supplementary Table 3 and Figures 3C,D). The results of this specific assay demonstrated the presence of genetic material from *Stenotrophomonas maltophilia* in BALF (Supplementary Table 3 and Figures 3C,D). Importantly, the concentration of *Stenotrophomonas maltophilia* DNA in the negative extraction controls was at a significantly lower level [mean (*SD*) PEG extraction controls: 2.26(3.2), mean (*SD*) PEG extracted samples: 765.57(784.49). Welch *t*-test *p*-value 0.01] (Supplementary Table 3). This data confirms that in our cohort, the OTU assigned to the *Stenotrophomonas* taxon is unlikely to be of contaminant origin, except for those extracts in which the quantity of bacterial DNA is similar to that of the negative extraction controls.

## Discussion

Microbiome research using low biomass samples requires special attention to prevent the introduction of unacceptable variability in the form of environmental contaminants (Salter et al., 2014). When the microbial density in the biological specimens is low, extraction procedures can easily introduce bias. This can impact true biological variability and seems to be dependent on available DNA concentration during analysis (Salter et al., 2014). To overcome this limitation, large volumes of starting material are often needed. This is not always possible, especially when dealing with bronchial washes from infants. In this work, we describe a new method for the isolation of DNA from low biomass BALF samples. We demonstrate that this novel protocol yields a higher number of 16S bacterial copies compared to the negative extraction control and captures a higher biological variability. Using equal-volume aliquots from the same BALF specimen, we also show that the PEG method performs better than a column-based kit specifically designed for microbiome studies in human body fluids.

Our study provides further “best practice” confirmation on the necessity of sequencing extraction negative controls, especially when using samples in which the expected microbial density is low (Eisenhofer et al., 2019). We observed in our study, that the taxonomic profiles of the BALF samples were dependent on the extraction method used. Absolute quantification of the bacterial DNA present in our extracts suggested that the contrasting OTU profiles were likely due to differences in the recovery of bacterial DNA associated with each extraction method. In light of these results we agree with recent global recommendation that, when possible, bacterial DNA quantification should be performed to discern true biological variation from environmental noise (Schneeberger et al., 2019).

In this study, our respiratory BALF samples were characterized by the presence of microorganisms associated with the oral cavity. A previous study has shown that oral flora, which can reach the lower airways through microaspiration, is associated with pulmonary inflammation (Segal et al., 2013; Dickson et al., 2017). In this regard, colonization of the lower airways by oral bacterial may underlie an important mechanism in airway disease progression. We noticed that most of the samples processed with the PEG method and especially the CF specimens, reported high levels of an OTU assigned to the *Stenotrophomonas* taxon. Since this organism is commonly identified as an environmental contaminant (Morris et al., 2013; Salter et al., 2014), we performed a species-specific absolute quantification assay. The results of this approach revealed a higher concentration (in some instances up to 1,000 times higher) of genetic material from *Stenotrophomonas maltophilia* in the biological specimens than in the negative controls, suggesting a true biological signal. Thus the higher detection of *Stenotrophomonas* in the PEG extracts is likely associated with the better performance shown by this protocol. *Stenotrophomonas maltophilia* represents an emergent pathogen in chronic lung disease, which is commonly detected in medical equipment in hospitals and as a transient colonizer in hospitalized patients (Conly and Shafran, 1996; Parkins and Floto, 2015). The association between the presence of this pathogen and the use of anti-*Pseudomonas* prophylaxis may also help to explain its prevalence in the OTU profiles of the CF cohort (Conly and Shafran, 1996; Parkins and Floto, 2015). However, because the absence of reported *Stenotrophomonas* infection in the study cohorts by classical clinical microbiological analysis, we recognize that this OTU could represent a transient colonizer. Further studies are required to ascertain if this observation with a possible emerging pathogen is biologically meaningful, especially in CF infants. On the basis of this observation, we also recommend that, apart from microbial load, quantification of suspected taxa through qPCR should be performed with microbiome studies.

We also provide two TaqMan^®^ assays based on those previously reported by Nadkarni et al. (2002) and Fraser et al. (2019). We redesigned the published probes to fit with the assay conditions recommended by Applied Biosystems, mainly related to the size of the targeted amplicon. Previous studies using BALF samples have quantified bacterial load through qPCR (Erb-Downward et al., 2011; May et al., 2015; Bernasconi et al., 2016; Laguna et al., 2016; Marsh et al., 2016; Zemanick et al., 2017; Pattaroni et al., 2018; Esther et al., 2019; Jorth et al., 2019; Schneeberger et al., 2019). In these reports, authors targeted DNA fragments of variable length ranging from 180 to 590 bp (Erb-Downward et al., 2011; May et al., 2015; Bernasconi et al., 2016; Laguna et al., 2016; Marsh et al., 2016; Zemanick et al., 2017; Pattaroni et al., 2018; Esther et al., 2019; Jorth et al., 2019; Schneeberger et al., 2019). Except for those primers used by Bernasconi et al. (2016) (180 bp), this amplicon length range is far from that recommended for qPCR (50–150 bp), because longer products show suboptimal amplification efficiencies (Debode et al., 2017). Unfortunately, any of those studies reported key experimental parameters recommended for publication of qPCR assays such as efficiency (Bustin et al., 2009). To ensure reproducibility and provide the reader with all the information necessary to evaluate the quality and interpretation of the data presented, we have described all the experimental information related to the redesigned TaqMan^®^ assays following the MIQE guidelines in Supplementary Table 8 (Bustin et al., 2009).

We acknowledge some limitations in our study. Firstly, we did not carry out a comprehensive comparison of different DNA extraction protocols. On the basis of the popularity of silica columns in microbiome studies using BALF (Supplementary Table 2), we only evaluated the performance of our PEG protocol with a column-based commercial kit. Thus, we cannot rule out that other extraction technologies may perform similarly to the PEG method. Secondly, we did not use communities with defined known composition. Thus, we cannot surely ensure the precision and dynamic range of the PEG protocol. Because of the former, we have been cautious of fitting all these limitations with the claims we have made.

In summary, we present a new method that compared to a commercial kit, increases the recovery of bacterial DNA from BALF samples. Although, we only tested this method using BALF material, this protocol could also be suitable for DNA isolation from different low-biomass biological specimens such as swabs. We are confident that this approach will further assist researchers to reveal the complex modalities of host-microbiota interaction in body niches composed of low microbial density and abundance.

## Data Availability Statement

The datasets presented in this study can be found in online repositories. The names of the repository/repositories and accession number(s) can be found below: https://www.ncbi.nlm.nih.gov/, Bioproject PRJNA636842.

## Ethics Statement

The studies involving human participants were reviewed and approved by the Princess Margaret Hospital for Children, Perth Ethics Committee (Ref. 1762/EPP; Date of approval December 10th, 2009) and Hunter New England LHD Ethics committee [Mechanisms of Inflammatory airways disease (05/08/10/3.09)]. Written informed consent to participate in this study was provided by the participants’ legal guardian/next of kin.

## Author Contributions

MS, JAC-M, and FO: conceptualization and writing–original draft preparation. MS and JAC-M: methodology, investigation, and visualization. JAC-M, MS, and PA-R: formal analysis. PABW and SMS: resources. JAC-M, MS, PA-R, PABW, SMS, and FO: writing–review and editing. All the authors approved the final version of the manuscript.

## Conflict of Interest

The authors declare that the research was conducted in the absence of any commercial or financial relationships that could be construed as a potential conflict of interest.

## Footnotes

1 http://www.arb-silva.de

## References

[B1] Aas J. A. Paster B. J. Stokes L. N. Olsen I. Dewhirst F. E. (2005). Defining the normal bacterial flora of the oral cavity. *J. Clin. Microbiol.* 43 5721–5732. 10.1128/JCM.43.11.5721-5732.2005 16272510 PMC1287824

[B2] Ahmed B. Cox M. J. Cuthbertson L. James P. L. Cookson W. O. C. Davies J. C. (2018). Comparison of the upper and lower airway microbiota in children with chronic lung diseases. *PLoS One* 13:e0201156. 10.1371/journal.pone.0201156 30071000 PMC6071972

[B3] Arbeli Z. Fuentes C. L. (2007). Improved purification and PCR amplification of DNA from environmental samples. *FEMS Microbiol. Lett.* 272 269–275. 10.1111/j.1574-6968.2007.00764.x 17521406

[B4] Bernasconi E. Pattaroni C. Koutsokera A. Pison C. Kessler R. Benden C. (2016). Airway microbiota determines innate cell inflammatory or tissue remodeling profiles in lung transplantation. *Am. J. Respir. Crit. Care Med.* 194 1252–1263. 10.1164/rccm.201512-2424OC 27248293

[B5] Bolger A. M. Lohse M. Usadel B. (2014). Trimmomatic: a flexible trimmer for Illumina sequence data. *Bioinformatics* 30 2114–2120. 10.1093/bioinformatics/btu170 24695404 PMC4103590

[B6] Borewicz K. Pragman A. A. Kim H. B. Hertz M. Wendt C. Isaacson R. E. (2013). Longitudinal analysis of the lung microbiome in lung transplantation. *FEMS Microbiol. Lett.* 339 57–65. 10.1111/1574-6968.12053 23173619 PMC3546157

[B7] Budden K. F. Gellatly S. L. Wood D. L. Cooper M. A. Morrison M. Hugenholtz P. (2017). Emerging pathogenic links between microbiota and the gut-lung axis. *Nat. Rev. Microbiol.* 15 55–63. 10.1038/nrmicro.2016.142 27694885

[B8] Budden K. F. Shukla S. D. Rehman S. F. Bowerman K. L. Keely S. Hugenholtz P. (2019). Functional effects of the microbiota in chronic respiratory disease. *Lancet Respir. Med.* 7 907–920. 10.1016/S2213-2600(18)30510-1 30975495

[B9] Bushnell B. Rood J. Singer E. (2017). BBMerge – accurate paired shotgun read merging via overlap. *PLoS One* 12:e0185056. 10.1371/journal.pone.0185056 29073143 PMC5657622

[B10] Bustin S. A. Benes V. Garson J. A. Hellemans J. Huggett J. Kubista M. (2009). The MIQE guidelines: minimum information for publication of quantitative real-time PCR experiments. *Clin. Chem.* 55 611–622. 10.1373/clinchem.2008.112797 19246619

[B11] Camacho C. Coulouris G. Avagyan V. Ma N. Papadopoulos J. Bealer K. (2009). BLAST+: architecture and applications. *BMC Bioinform.* 10:421. 10.1186/1471-2105-10-421 20003500 PMC2803857

[B12] Caparros-Martin J. A. Flynn S. Reen F. J. Woods D. F. Agudelo-Romero P. Ranganathan S. C. (2020). The detection of bile acids in the lungs of paediatric cystic fibrosis patients is associated with altered inflammatory patterns. *Diagnostics* 10:282. 10.3390/diagnostics10050282 32384684 PMC7277992

[B13] Castro-Mejia J. L. Muhammed M. K. Kot W. Neve H. Franz C. M. Hansen L. H. (2015). Optimizing protocols for extraction of bacteriophages prior to metagenomic analyses of phage communities in the human gut. *Microbiome* 3:64. 10.1186/s40168-015-0131-4 26577924 PMC4650499

[B14] Champely S. (2018). *PairedData: Paired Data Analysis. R Package Version 1.1.1.* Available online at: https://CRAN.R-project.org/package=PairedData (accessed April, 2020).

[B15] Charlson E. S. Bittinger K. Haas A. R. Fitzgerald A. S. Frank I. Yadav A. (2011). Topographical continuity of bacterial populations in the healthy human respiratory tract. *Am. J. Respir. Crit. Care Med.* 184 957–963. 10.1164/rccm.201104-0655OC 21680950 PMC3208663

[B16] Cheng C. Jia J. L. Ran S. Y. (2015). Polyethylene glycol and divalent salt-induced DNA reentrant condensation revealed by single molecule measurements. *Soft Matter* 11 3927–3935. 10.1039/c5sm00619h 25871460

[B17] Cho I. Blaser M. J. (2012). The human microbiome: at the interface of health and disease. *Nat. Rev. Genet.* 13 260–270. 10.1038/nrg3182 22411464 PMC3418802

[B18] Conly J. Shafran S. (1996). Pseudo-, Xantho-, and now *Stenotrophomonas* maltophilia: new kid on the block. *Can. J. Infect. Dis.* 7 99–100. 10.1155/1996/585141 22514424 PMC3327390

[B19] Davis N. M. Proctor D. M. Holmes S. P. Relman D. A. Callahan B. J. (2018). Simple statistical identification and removal of contaminant sequences in marker-gene and metagenomics data. *Microbiome* 6:226. 10.1186/s40168-018-0605-2 30558668 PMC6298009

[B20] Debode F. M. A. Janssen E. Bragard C. Berben G. (2017). The influence of amplicon length on real-time PCR results. *Biotechnol. Agron. Soc. Environ.* 21 3–11. 10.25518/1780-4507.13461

[B21] Deregibus M. C. Figliolini F. D’Antico S. Manzini P. M. Pasquino C. De Lena M. (2016). Charge-based precipitation of extracellular vesicles. *Int. J. Mol. Med.* 38 1359–1366. 10.3892/ijmm.2016.2759 28025988 PMC5065305

[B22] Dickson R. P. Erb-Downward J. R. Freeman C. M. McCloskey L. Beck J. M. Huffnagle G. B. (2015). Spatial variation in the healthy human lung microbiome and the adapted island model of lung biogeography. *Ann. Am. Thorac. Soc.* 12 821–830. 10.1513/AnnalsATS.201501-029OC 25803243 PMC4590020

[B23] Dickson R. P. Erb-Downward J. R. Freeman C. M. McCloskey L. Falkowski N. R. Huffnagle G. B. (2017). Bacterial topography of the healthy human lower respiratory tract. *mBio* 8:e02287-16. 10.1128/mBio.02287-16 28196961 PMC5312084

[B24] Dickson R. P. Erb-Downward J. R. Martinez F. J. Huffnagle G. B. (2016a). The microbiome and the respiratory tract. *Annu. Rev. Physiol.* 78 481–504. 10.1146/annurev-physiol-021115-105238 26527186 PMC4751994

[B25] Dickson R. P. Singer B. H. Newstead M. W. Falkowski N. R. Erb-Downward J. R. Standiford T. J. (2016b). Enrichment of the lung microbiome with gut bacteria in sepsis and the acute respiratory distress syndrome. *Nat. Microbiol.* 1:16113. 10.1038/nmicrobiol.2016.113 27670109 PMC5076472

[B26] Eisenhofer R. Minich J. J. Marotz C. Cooper A. Knight R. Weyrich L. S. (2019). Contamination in low microbial biomass microbiome studies: issues and recommendations. *Trends Microbiol.* 27 105–117. 10.1016/j.tim.2018.11.003 30497919

[B27] Erb-Downward J. R. Thompson D. L. Han M. K. Freeman C. M. McCloskey L. Schmidt L. A. (2011). Analysis of the lung microbiome in the “healthy” smoker and in COPD. *PLoS One* 6:e16384. 10.1371/journal.pone.0016384 21364979 PMC3043049

[B28] Esther C. R. Jr. Muhlebach M. S. Ehre C. Hill D. B. Wolfgang M. C. Kesimer M. (2019). Mucus accumulation in the lungs precedes structural changes and infection in children with cystic fibrosis. *Sci. Transl. Med.* 11:eaav3488. 10.1126/scitranslmed.aav3488 30944166 PMC6566903

[B29] Ewels P. Magnusson M. Lundin S. Kaller M. (2016). MultiQC: summarize analysis results for multiple tools and samples in a single report. *Bioinformatics* 32 3047–3048. 10.1093/bioinformatics/btw354 27312411 PMC5039924

[B30] Faner R. Sibila O. Agusti A. Bernasconi E. Chalmers J. D. Huffnagle G. B. (2017). The microbiome in respiratory medicine: current challenges and future perspectives. *Eur. Respir. J.* 49:1602086. 10.1183/13993003.02086-2016 28404649

[B31] Fraser T. A. Bell M. G. Harris P. N. A. Bell S. C. Bergh H. Nguyen T. K. (2019). Quantitative real-time PCR assay for the rapid identification of the intrinsically multidrug-resistant bacterial pathogen *Stenotrophomonas* maltophilia. *Microb. Genomics* 5:e000307. 10.1099/mgen.0.000307 31617838 PMC6861864

[B32] Frayman K. B. Armstrong D. S. Carzino R. Ferkol T. W. Grimwood K. Storch G. A. (2017). The lower airway microbiota in early cystic fibrosis lung disease: a longitudinal analysis. *Thorax* 72 1104–1112. 10.1136/thoraxjnl-2016-209279 28280235

[B33] Garcia-Romero N. Madurga R. Rackov G. Palacin-Aliana I. Nunez-Torres R. Asensi-Puig A. (2019). Polyethylene glycol improves current methods for circulating extracellular vesicle-derived DNA isolation. *J. Transl. Med.* 17:75. 10.1186/s12967-019-1825-3 30871557 PMC6419425

[B34] Gomes S. Cavadas B. Ferreira J. C. Marques P. I. Monteiro C. Sucena M. (2019). Profiling of lung microbiota discloses differences in adenocarcinoma and squamous cell carcinoma. *Sci. Rep.* 9:12838. 10.1038/s41598-019-49195-w 31492894 PMC6731246

[B35] Han M. K. Zhou Y. Murray S. Tayob N. Noth I. Lama V. N. (2014). Lung microbiome and disease progression in idiopathic pulmonary fibrosis: an analysis of the COMET study. *Lancet Respir. Med.* 2 548–556. 10.1016/S2213-2600(14)70069-4 24767767 PMC4142525

[B36] Jorth P. Ehsan Z. Rezayat A. Caldwell E. Pope C. Brewington J. J. (2019). Direct lung sampling indicates that established pathogens dominate early infections in children with cystic fibrosis. *Cell Rep.* 27 1190–1204.e3. 10.1016/j.celrep.2019.03.086 31018133 PMC6668708

[B37] Kalarikkal S. P. Prasad D. Kasiappan R. Chaudhari S. R. Sundaram G. M. (2020). A cost-effective polyethylene glycol-based method for the isolation of functional edible nanoparticles from ginger rhizomes. *Sci. Rep.* 10:4456. 10.1038/s41598-020-61358-8 32157137 PMC7064537

[B38] Kloepfer K. M. Deschamp A. R. Ross S. E. Peterson-Carmichael S. L. Hemmerich C. M. Rusch D. B. (2018). In children, the microbiota of the nasopharynx and bronchoalveolar lavage fluid are both similar and different. *Pediatr. Pulmonol.* 53 475–482. 10.1002/ppul.23953 29405661 PMC6542268

[B39] Knight R. Callewaert C. Marotz C. Hyde E. R. Debelius J. W. McDonald D. (2017). The microbiome and human biology. *Annu. Rev. Genomics Hum. Genet.* 18 65–86. 10.1146/annurev-genom-083115-022438 28375652

[B40] Kyo M. Nishioka K. Nakaya T. Kida Y. Tanabe Y. Ohshimo S. (2019). Unique patterns of lower respiratory tract microbiota are associated with inflammation and hospital mortality in acute respiratory distress syndrome. *Respir. Res.* 20:246. 10.1186/s12931-019-1203-y 31694652 PMC6836399

[B41] Laguna T. A. Wagner B. D. Williams C. B. Stevens M. J. Robertson C. E. Welchlin C. W. (2016). Airway microbiota in bronchoalveolar lavage fluid from clinically well infants with cystic fibrosis. *PLoS One* 11:e0167649. 10.1371/journal.pone.0167649 27930727 PMC5145204

[B42] Le Cao K. A. Costello M. E. Lakis V. A. Bartolo F. Chua X. Y. Brazeilles R. (2016). MixMC: a multivariate statistical framework to gain insight into microbial communities. *PLoS One* 11:e0160169. 10.1371/journal.pone.0160169 27513472 PMC4981383

[B43] Lokwani R. Wark P. A. B. Baines K. J. Barker D. Simpson J. L. (2019). Hypersegmented airway neutrophils and its association with reduced lung function in adults with obstructive airway disease: an exploratory study. *BMJ Open* 9:e024330. 10.1136/bmjopen-2018-024330 30696679 PMC6352776

[B44] Marchesi J. R. Sato T. Weightman A. J. Martin T. A. Fry J. C. Hiom S. J. (1998). Design and evaluation of useful bacterium-specific PCR primers that amplify genes coding for bacterial 16S rRNA. *Appl. Environ. Microbiol.* 64 795–799. 9464425 10.1128/aem.64.2.795-799.1998 PMC106123

[B45] Marsh R. L. Kaestli M. Chang A. B. Binks M. J. Pope C. E. Hoffman L. R. (2016). The microbiota in bronchoalveolar lavage from young children with chronic lung disease includes taxa present in both the oropharynx and nasopharynx. *Microbiome* 4:37. 10.1186/s40168-016-0182-1 27388563 PMC4936249

[B46] Marsland B. J. Trompette A. Gollwitzer E. S. (2015). The gut-lung axis in respiratory disease. *Ann. Am. Thorac. Soc.* 12(Suppl. 2) S150–S156. 10.1513/AnnalsATS.201503-133AW 26595731

[B47] May A. K. Brady J. S. Romano-Keeler J. Drake W. P. Norris P. R. Jenkins J. M. (2015). A pilot study of the noninvasive assessment of the lung microbiota as a potential tool for the early diagnosis of ventilator-associated pneumonia. *Chest* 147 1494–1502. 10.1378/chest.14-1687 25474571 PMC4451706

[B48] Morris A. Beck J. M. Schloss P. D. Campbell T. B. Crothers K. Curtis J. L. (2013). Comparison of the respiratory microbiome in healthy nonsmokers and smokers. *Am. J. Respir. Crit. Care Med.* 187 1067–1075. 10.1164/rccm.201210-1913OC 23491408 PMC3734620

[B49] Mott L. S. Park J. Murray C. P. Gangell C. L. de Klerk N. H. Robinson P. J. (2012). Progression of early structural lung disease in young children with cystic fibrosis assessed using CT. *Thorax* 67 509–516. 10.1136/thoraxjnl-2011-200912 22201161

[B50] Nadkarni M. A. Martin F. E. Jacques N. A. Hunter N. (2002). Determination of bacterial load by real-time PCR using a broad-range (universal) probe and primers set. *Microbiology* 148(Pt 1) 257–266. 10.1099/00221287-148-1-257 11782518

[B51] Oksanen J. Blanchet F. G. Friendly M. Kindt R. Legendre P. McGlinn D. (2016). *vegan**: Community Ecology Package. R package version 2.4-0.* Available online at: https://CRAN.R-project.org/package=vegan (accessed April, 2020).

[B52] Parkins M. D. Floto R. A. (2015). Emerging bacterial pathogens and changing concepts of bacterial pathogenesis in cystic fibrosis. *J. Cystic Fibrosis* 14 293–304. 10.1016/j.jcf.2015.03.012 25881770

[B53] Pattaroni C. Watzenboeck M. L. Schneidegger S. Kieser S. Wong N. C. Bernasconi E. (2018). Early-life formation of the microbial and immunological environment of the human airways. *Cell Host Microbe* 24 857–65e4. 10.1016/j.chom.2018.10.019 30503510

[B54] Pragman A. A. Kim H. B. Reilly C. S. Wendt C. Isaacson R. E. (2012). The lung microbiome in moderate and severe chronic obstructive pulmonary disease. *PLoS One* 7:e47305. 10.1371/journal.pone.0047305 23071781 PMC3469539

[B55] Quast C. Pruesse E. Yilmaz P. Gerken J. Schweer T. Yarza P. (2013). The SILVA ribosomal RNA gene database project: improved data processing and web-based tools. *Nucleic Acids Res.* 41 D590–D596. 10.1093/nar/gks1219 23193283 PMC3531112

[B56] R Core Team, (2017). *R: A Language and Environment for Statistical Computing.* Vienna: R Foundation for Statistical Computing.

[B57] Renwick J. McNally P. John B. DeSantis T. Linnane B. Murphy P. (2014). The microbial community of the cystic fibrosis airway is disrupted in early life. *PLoS One* 9:e109798. 10.1371/journal.pone.0109798 25526264 PMC4272276

[B58] Rohart F. Gautier B. Singh A. Le Cao K. A. (2017). mixOmics: an R package for ’omics feature selection and multiple data integration. *PLoS Comput. Biol.* 13:e1005752. 10.1371/journal.pcbi.1005752 29099853 PMC5687754

[B59] Salter S. J. Cox M. J. Turek E. M. Calus S. T. Cookson W. O. Moffatt M. F. (2014). Reagent and laboratory contamination can critically impact sequence-based microbiome analyses. *BMC Biol.* 12:87. 10.1186/s12915-014-0087-z 25387460 PMC4228153

[B60] Schneeberger P. H. H. Prescod J. Levy L. Hwang D. Martinu T. Coburn B. (2019). Microbiota analysis optimization for human bronchoalveolar lavage fluid. *Microbiome* 7:141. 10.1186/s40168-019-0755-x 31665066 PMC6821041

[B61] Segal L. N. Alekseyenko A. V. Clemente J. C. Kulkarni R. Wu B. Gao Z. (2013). Enrichment of lung microbiome with supraglottic taxa is associated with increased pulmonary inflammation. *Microbiome* 1:19. 10.1186/2049-2618-1-19 24450871 PMC3971609

[B62] Tighe S. Afshinnekoo E. Rock T. M. McGrath K. Alexander N. McIntyre A. (2017). Genomic methods and microbiological technologies for profiling novel and extreme environments for the extreme microbiome project (XMP). *J. Biomol. Tech.* 28 31–39. 10.7171/jbt.17-2801-004 28337070 PMC5345951

[B63] Tong X. Su F. Xu X. Xu H. Yang T. Xu Q. (2019). Alterations to the lung microbiome in idiopathic pulmonary fibrosis patients. *Front. Cell. Infect. Microbiol.* 9:149. 10.3389/fcimb.2019.00149 31165050 PMC6536613

[B64] van de Guchte M. Blottiere H. M. Dore J. (2018). Humans as holobionts: implications for prevention and therapy. *Microbiome* 6:81. 10.1186/s40168-018-0466-8 29716650 PMC5928587

[B65] Wang H. Zhou Q. Dai W. Feng X. Lu Z. Yang Z. (2019). Lung microbiota and pulmonary inflammatory cytokines expression vary in children with tracheomalacia and adenoviral or mycoplasma pneumoniae pneumonia. *Front. Pediatr.* 7:265. 10.3389/fped.2019.00265 31316955 PMC6611399

[B66] Wen Y. Xiao F. Wang C. Wang Z. (2016). The impact of different methods of DNA extraction on microbial community measures of BALF samples based on metagenomic data. *Am. J. Transl. Res.* 8 1412–1425. 27186268 PMC4858570

[B67] Wickham H. (2016). *ggplot2: Elegant Graphics for Data Analysis.* New York: Springer-Verlag.

[B68] Willner D. Daly J. Whiley D. Grimwood K. Wainwright C. E. Hugenholtz P. (2012). Comparison of DNA extraction methods for microbial community profiling with an application to pediatric bronchoalveolar lavage samples. *PLoS One* 7:e34605. 10.1371/journal.pone.0034605 22514642 PMC3326054

[B69] Zemanick E. T. Wagner B. D. Robertson C. E. Ahrens R. C. Chmiel J. F. Clancy J. P. (2017). Airway microbiota across age and disease spectrum in cystic fibrosis. *Eur. Respir. J.* 50:1700832. 10.1183/13993003.00832-2017 29146601 PMC5935257

