# Supplementary material for: Microbiomic Analysis on Low Abundant Respiratory Biomass Samples; Improved Recovery of Microbial DNA From Bronchoalveolar Lavage Fluid
Source: Front Microbiol. 2020 Oct 6;11:572504. doi: 10.3389/fmicb.2020.572504 (PMC7573210; doi:10.3389/fmicb.2020.572504)
Supplement: Supplementary file 1 [file Data_Sheet_1.ZIP › SALADIE_SUPPMATERIAL.docx]

**SUPPLEMENTAL MATERIAL**

MICROBIOMIC ANALYSIS ON LOW ABUNDANT RESPIRATORY BIOMASS SAMPLES; IMPROVED RECOVERY OF MICROBIAL DNA FROM BRONCHOALVEOLAR LAVAGE FLUID

Montserrat Saladié, Jose Antonio Caparrós-Martín, Patricia Agudelo-Romero, Peter AB Wark, Stephen M Stick and Fergal O’Gara

**Supplemental Figure 1**

**Supplemental figure 1. DNA extraction workflow using the PEG-based protocol described in this paper.**


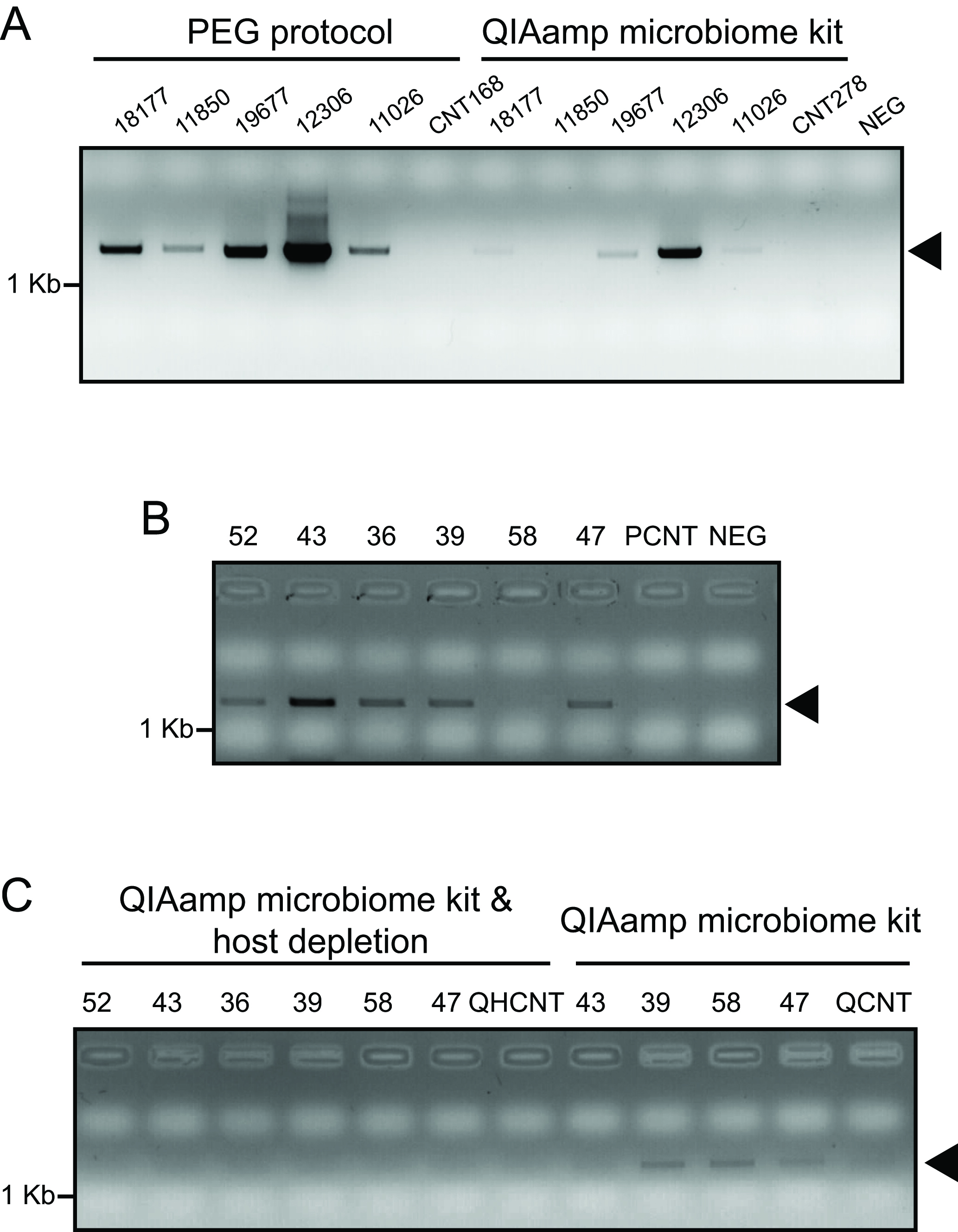
**Supplemental Figure 2**

**Supplemental figure 2. PCR analysis of the DNA extracts. A-C.** PCR-mediated amplification of a 1344 bp DNA fragment from the *16S rRNA* gene using the primers 63F and 1387R ([1](#_ENREF_1)). Arrowheads point to the amplification of a single specific product of the expected size. DNA extracts were obtained from the COPD (A) and CF (B-C) BALF samples using the PEG protocol (A-B) or the QIAamp microbiome kit (A,C) with or without host depletion as indicated. Each lane is identified with a numeric code that identifies each BALF biological specimen. Each set of samples was run next to its corresponding negative extraction control. Negative extraction controls are indicated with the following alphanumeric codes: CNT168, CNT278, PCNT, QHCNT, QCNT. No template PCR negative controls are indicated with NEG.

**Supplemental Figure 3**

**
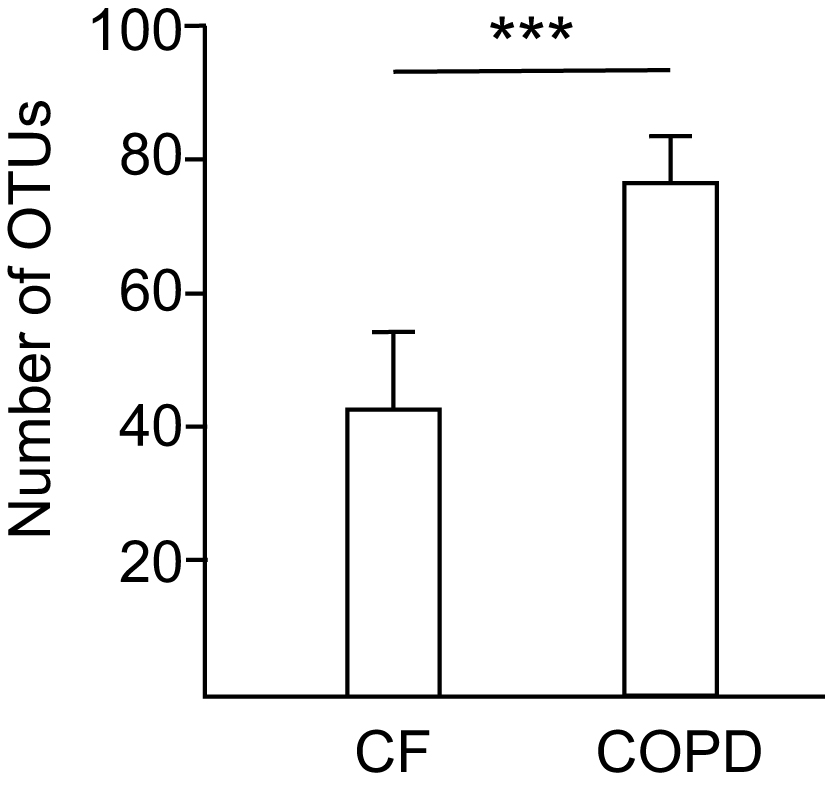
**

**Supplemental figure 3.** Number of OTUs (richness) in the CF and COPD BALF samples extracted with the PEG protocol. Welch t-test was used for statistical inference. ***, *P* <0.001.

**Supplemental Figure 4**

**
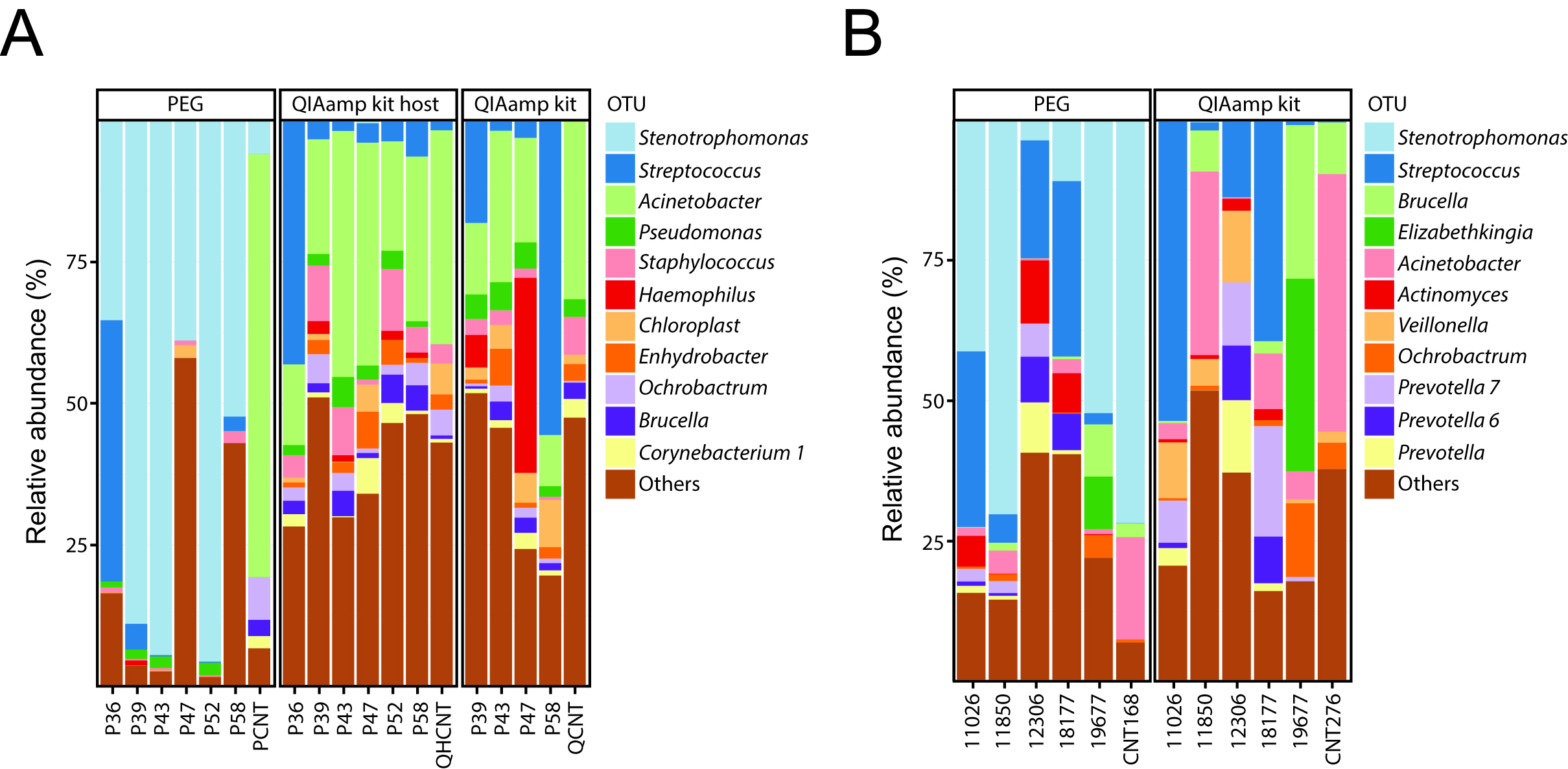
**

**Supplemental figure 4. Taxonomic profiles of the BALF-associated microbial communities. A-B.** Relative microbial taxonomic composition in the CF (A) and COPD (B) cohort.

**Supplemental Table 1.** Methodological strategies for isolating bacterial DNA from BALF biological specimens obtained after a literature survey. Volume of BALF used is only included when it was clearly specified in the corresponding material and methods section. * The publication by Zemanick and colleagues in 2017 reported the employment of 0.2 µL of BALF as starting material, which could be a typographical error ([2](#_ENREF_2)). AR, age range; SD, Standard deviation; IQR, interquartile range.

| Protocol | Volume of BALF (µL) | Age (years) | Reference |
| --- | --- | --- | --- |
| QIAamp DNA Mini Kit (QIAGEN) | Not specified | AR: 0-0.16 | ([3](#_ENREF_3)) |
| cetyl trimethylammonium bromide protocol | 400 | AR: 4-6 | ([4](#_ENREF_4)) |
| High saline protocol | 400 | AR: 4-6 | ([4](#_ENREF_4)) |
| Nucleospin Tissue Kit (Macherey-Nagel) | 400 | AR: 4-6 | ([4](#_ENREF_4)) |
| MoBio PowerSoil DNA Isolation Kit (MoBio) | 400 | AR: 4-6 | ([4](#_ENREF_4)) |
| Bead beating pre-treatment and QIAamp columns (QIAgen) | 200 | ≤10 | ([5](#_ENREF_5)) |
| Qiagen EZ1 Advanced automated extraction platform (QIAGEN) | 0.2^*^ | AR: 0.16-50 | ([2](#_ENREF_2)) |
| Qiagen EZ1 Advanced automated extraction platform (QIAGEN) | Not specified | AR: 0.6-1 | ([6](#_ENREF_6)) |
| Bead beating pre-treatment and Qiagen DNeasyBlood & Tissue kit (QIAGEN) | Not specified | AR: 18-77 | ([7](#_ENREF_7)) |
| Bead beating pre-treatment and Qiagen DNeasyBlood & Tissue kit (QIAGEN) | 5000 | AR: 35-80 | ([8](#_ENREF_8)) |
| Roche MangNA Pure Compact System Automated DNA Extraction (Roche) | Not specified | Not specified  Mean(SD): 3.3(1.7) and 3.2(2) | ([9](#_ENREF_9)) |
| Bead beating pre-treatment and MagNA Pure Compact system and Nucleic Acid Isolation Kit I (Roche) | 5000 | AR: 40-78 | ([10](#_ENREF_10)) |
| QIAamp DNA Mini Kit (QIAGEN) | Not specified | Not specified | ([11](#_ENREF_11)) |
| NUCLISENS® EASYMAG® ( bioMérieux) | 200 | AR: 0.1-6.5 | ([12](#_ENREF_12)) |
| Qiagen DNeasy Blood & Tissue Kit (QIAGEN) | 1000 | Median 3.3, IQR 0.25-18.3 | ([13](#_ENREF_13)) |
| Bead beating pre-treatment, chloroform extraction and ethanol:linear acrylamide solution for DNA precipitation | 100 | AR: 6-21 | ([14](#_ENREF_14)) |
| Bead beating pre-treatment and isopropanol for DNA precipitation | 500 | AR: 25-78 | ([15](#_ENREF_15)) |
| MPBio FastDNA Spin Kit for Soil | 500-4000 | AR: 0.1-16.2 | ([16](#_ENREF_16)) |
| DNeasy Blood & Tissue Kit (QIAGEN) | Not specified | Not specified | ([17](#_ENREF_17)) |
| PowerSoil DNA isolation kit (Mo Bio) | 250 | Not specified | ([18](#_ENREF_18)) |
| Power Soil DNA Isolation Kit (Mo Bio) | Not specified | AR: 0.3-9.6 | ([19](#_ENREF_19)) |
| NucleoSpin Microbial DNA (Macherey-Nagel) | Not specified | Median 67, IQR 59-77; median 67, IQR 60-77; median 67, IQR 57-80 | ([20](#_ENREF_20)) |
| Different column-based kits (QIAGEN) | 3000 | Mean (SD): 60.58 (12.82) | ([21](#_ENREF_21)) |
| PowerSoil DNA isolation kit (MoBio) | 1800 | AR: 31-65 | ([22](#_ENREF_22)) |
| DNA Mini kit (Qiagen) | 200 | AR: 31-89 | ([23](#_ENREF_23)) |
| Bead beating pre-treatment and isopropanol for DNA precipitation | 750 | Not specified | ([24](#_ENREF_24)) |
| QIAamp DNA Microbiome Kit (QIAGEN) | 3000 | Mean (SD): 62.71(7.9) and 61.28(9.37) | ([25](#_ENREF_25)) |
| Bead beating pre-treatment and Qiagen DNA/RNA AllPrep Mini kit (QIAGEN) | Not specified | AR: 0.9-9.75 | ([26](#_ENREF_26)) |

**Supplemental Table 2.** Clinical information of the patients enrolled in this study. COPD subjects are classified accordingly to The Global Initiative for Chronic Obstructive Lung Disease (GOLD) guidelines for COPD. CFU, colony forming units; CF, cystic fibrosis; HS, healthy smoker; COPD, chronic obstructive pulmonary disease; RML, right middle lobe; MOF, mixed oral flora; *E. coli*, *Escherichia coli*; *S. aureus*, *Staphylococcus aureus*; HPIV, human parainfluenza virus; RSV, Respiratory syncytial virus; *E. Sp*, *Enterobacter sp*; *K. sp*, *Klebsiella sp.* * In the COPD cohort, clinical microbiology was reported as qualitative data.

| Patient ID | Disease | Age (years) | Lobe sampled | Clinical microbiology (CFU)* |
| --- | --- | --- | --- | --- |
| P52 | CF | 0.3 | RML | MOF (1,000,000) |
| P36 | CF | 0.28 | RML | *E.coli* (1,000,000), MOF (1,000,000) *S. aureus* (1,000,000) |
| P39 | CF | 0.55 | RML | MOF (10,000,000), Positive for HPIV |
| P43 | CF | 0.71 | RML | Positive for RSV |
| P47 | CF | 0.83 | RML | None detected |
| P58 | CF | 0.91 | RML | *E. sp* (1,000,000), *K. sp* (1,000,000), MOF (10,000,000) |
| 11850 | HS | 48 | RML | None detected |
| 18177 | COPD-GOLD1 | 45 | RML | None detected |
| 12306 | COPD-GOLD2 | 82 | RML | None detected |
| 11026 | COPD-GOLD3 | 76 | RML | *Pseudomonas aeruginosa* |
| 19677 | COPD-GOLD4 | 55 | RML | None detected |

**Supplemental Table 3.** Molecular characterization of the DNA extracts reported in this study. CF, cystic fibrosis; HS, healthy smoker; COPD, chronic obstructive pulmonary disease; NEC, negative extraction control; PEG, polyethylene glycol; Q, QIAamp microbiome kit; QH, QIAamp microbiome kit with DNA host depletion; <LOD, under the limit of detection. Missing values are represented with NA. For those samples labelled as NAs, there was not enough DNA extract to perform the *Stenotrophomonas maltophilia*-specific qPCR.

| Sample ID | Sample group | Extraction method | DNA concentration (ng µL^-1^) | Total bacterial load (16S copies µL^-1^ of DNA extract) | *Stenotrophomonas maltophilia* load (16S copies µL-1 of DNA extract) |
| --- | --- | --- | --- | --- | --- |
| P52 | CF patient | PEG | 26.5 | 33,237.47 | 7,736.37 |
| P36 | CF patient | PEG | 8.1 | 4,902.77 | 322.09 |
| P39 | CF patient | PEG | 6.1 | 4,363.32 | 663.02 |
| P43 | CF patient | PEG | 66.9 | 20,093.52 | 5,391.15 |
| P47 | CF patient | PEG | 4.8 | 676.91 (<LOD) | 29.6 (<LOD) |
| P58 | CF patient | PEG | 7.3 | 637.58 (<LOD) | 7.9 (<LOD) |
| PCNT | NEC | PEG | -0.9 | 872.86 | 0 |
| QH52 | CF patient | QH | 0.8 | 935.37 | 0 |
| QH36 | CF patient | QH | 2.2 | 1,104.8 | 0 |
| QH39 | CF patient | QH | 1 | 629.04 (<LOD) | 5.5 (<LOD) |
| QH43 | CF patient | QH | 0.5 | 838.72 | NA |
| QH47 | CF patient | QH | 2.1 | 955.01 | 0.9 (<LOD) |
| QH58 | CF patient | QH | 1.9 | 965.13 | 5.4 (<LOD) |
| QHCNT | NEC | QH | 0.2 | 980.4 | 0 |
| QO39 | CF patient | Q | 6.8 | 1,183.78 | 0 |
| QO43 | CF patient | Q | 23.3 | 1,200.3 | 16.7 (<LOD) |
| QO47 | CF patient | Q | 2.4 | 1,317.56 | 5.7 (<LOD) |
| QO58 | CF patient | Q | 5.6 | 1,158.39 | 0 |
| QCNT | NEC | Q | 2 | 1,038.8 | 0 |
| 11850 | HS | PEG | 9.2 | 7,601.55 | 1,030.28 |
| 18177 | COPD-GOLD1 | PEG | 9.3 | 6,014.55 | NA |
| 12306 | COPD-GOLD2 | PEG | 19 | 7,991.1 | 2,545 |
| 11026 | COPD-GOLD3 | PEG | 16.4 | 21,808.75 | 7,503.9 |
| 19677 | COPD-GOLD4 | PEG | 128 | 26,514.93 | 5,393.5 |
| CNT168 | NEC | PEG | 1.8 | 649.38 (<LOD) | 18.1 (<LOD) |
| Q11850 | HS | Q | -1.3 | 1,418.67 | 0 |
| Q18177 | COPD-GOLD1 | Q | 0.9 | 1,026.06 | 0 |
| Q12306 | COPD-GOLD2 | Q | 0.9 | 2,984.12 | 0 |
| Q11026 | COPD-GOLD3 | Q | -0.9 | 2,160.65 | 0 |
| Q19677 | COPD-GOLD4 | Q | 7 | 3,644.25 | 0 |
| CNT278 | NEC | Q | -1.4 | 1,635.11 | 0 |

**Supplemental Table 4.** Contaminant taxa detected in the negative extraction controls. PCNT and CNT168 are negative extraction controls using the PEG protocol. QHCNT, QCNT and CNT278 are negative extraction controls associated with extractions using the QIAamp microbiome kit with or without host depletion. OTU, Operational Taxonomic Unit.

| **OTU abundance** | | | | |  |  |
| --- | --- | --- | --- | --- | --- | --- |
| **PCNT** | **QHCNT** | **QCNT** | **CNT168** | **CNT278** | **OTU** |  |
| 0 | 124 | 0 | 0 | 0 | Bacteria;Acidobacteria;Blastocatellia (Subgroup 4);Pyrinomonadales;Pyrinomonadaceae;RB41; |  |
| 0 | 1 | 0 | 0 | 0 | Bacteria;Acidobacteria;Subgroup 6; | |
| 0 | 0 | 0 | 1 | 0 | Bacteria;Actinobacteria;Actinobacteria;Actinomycetales;Actinomycetaceae;Actinomyces; | |
| 0 | 75 | 4 | 0 | 0 | Bacteria;Actinobacteria;Actinobacteria;Bifidobacteriales;Bifidobacteriaceae;Bifidobacterium; | |
| 232 | 162 | 540 | 187 | 322 | Bacteria;Actinobacteria;Actinobacteria;Corynebacteriales;Corynebacteriaceae;Corynebacterium 1; | |
| 0 | 391 | 1 | 0 | 0 | Bacteria;Actinobacteria;Actinobacteria;Corynebacteriales;Corynebacteriaceae;Corynebacterium; | |
| 0 | 0 | 0 | 48 | 0 | Bacteria;Actinobacteria;Actinobacteria;Corynebacteriales;Corynebacteriaceae;Lawsonella; | |
| 0 | 2 | 0 | 0 | 0 | Bacteria;Actinobacteria;Actinobacteria;Micrococcales;Brevibacteriaceae;Brevibacterium; | |
| 0 | 1 | 0 | 0 | 0 | Bacteria;Actinobacteria;Actinobacteria;Micrococcales;Microbacteriaceae;Microbacterium; | |
| 175 | 0 | 81 | 191 | 25 | Bacteria;Actinobacteria;Actinobacteria;Micrococcales;Micrococcaceae;Arthrobacter; | |
| 1 | 0 | 560 | 0 | 1 | Bacteria;Actinobacteria;Actinobacteria;Micrococcales;Micrococcaceae;Kocuria; | |
| 0 | 464 | 0 | 0 | 1 | Bacteria;Actinobacteria;Actinobacteria;Micrococcales;Micrococcaceae;Micrococcus; | |
| 0 | 95 | 0 | 3 | 2 | Bacteria;Actinobacteria;Actinobacteria;Micrococcales;Micrococcaceae;Rothia; | |
| 1 | 585 | 321 | 0 | 161 | Bacteria;Actinobacteria;Actinobacteria;Propionibacteriales;Propionibacteriaceae;Cutibacterium; | |
| 0 | 0 | 0 | 3 | 0 | Bacteria;Actinobacteria;Actinobacteria;Streptomycetales;Streptomycetaceae;Streptomyces; | |
| 0 | 1 | 0 | 0 | 0 | Bacteria;Actinobacteria;Coriobacteriia;Coriobacteriales;Atopobiaceae;Atopobium; | |
| 17 | 31 | 43 | 29 | 23 | Bacteria;Bacteroidetes;Bacteroidia;Bacteroidales;Bacteroidaceae;Bacteroides; | |
| 0 | 7 | 0 | 0 | 0 | Bacteria;Bacteroidetes;Bacteroidia;Bacteroidales;Dysgonomonadaceae;Proteiniphilum; | |
| 425 | 1 | 0 | 0 | 1 | Bacteria;Bacteroidetes;Bacteroidia;Bacteroidales;Muribaculaceae; | |
| 0 | 0 | 0 | 3 | 0 | Bacteria;Bacteroidetes;Bacteroidia;Bacteroidales;Porphyromonadaceae;Porphyromonas; | |
| 0 | 7 | 0 | 1 | 0 | Bacteria;Bacteroidetes;Bacteroidia;Bacteroidales;Prevotellaceae;Alloprevotella; | |
| 0 | 0 | 1 | 0 | 0 | Bacteria;Bacteroidetes;Bacteroidia;Bacteroidales;Prevotellaceae;Prevotella 2; | |
| 3 | 0 | 1 | 0 | 2 | Bacteria;Bacteroidetes;Bacteroidia;Bacteroidales;Prevotellaceae;Prevotella 6; | |
| 9 | 5 | 0 | 1 | 0 | Bacteria;Bacteroidetes;Bacteroidia;Bacteroidales;Prevotellaceae;Prevotella 7; | |
| 2 | 0 | 0 | 0 | 1 | Bacteria;Bacteroidetes;Bacteroidia;Bacteroidales;Prevotellaceae;Prevotella; | |
| 0 | 2 | 0 | 0 | 0 | Bacteria;Bacteroidetes;Bacteroidia;Bacteroidales;Prevotellaceae;Prevotellaceae UCG-001; | |
| 4 | 180 | 4 | 7 | 1 | Bacteria;Bacteroidetes;Bacteroidia;Bacteroidales;Rikenellaceae;Alistipes; | |
| 0 | 1 | 1 | 0 | 0 | Bacteria;Bacteroidetes;Bacteroidia;Chitinophagales;Chitinophagaceae;Terrimonas; | |
| 0 | 0 | 1 | 1 | 0 | Bacteria;Bacteroidetes;Bacteroidia;Chitinophagales;Saprospiraceae;uncultured; | |
| 1 | 0 | 0 | 0 | 197 | Bacteria;Bacteroidetes;Bacteroidia;Flavobacteriales;Flavobacteriaceae;Flavobacterium; | |
| 0 | 72 | 0 | 6 | 0 | Bacteria;Bacteroidetes;Bacteroidia;Flavobacteriales;Weeksellaceae;Chryseobacterium; | |
| 0 | 4 | 0 | 2 | 0 | Bacteria;Bacteroidetes;Bacteroidia;Flavobacteriales;Weeksellaceae;Elizabethkingia; | |
| 0 | 0 | 0 | 117 | 485 | Bacteria;Bacteroidetes;Bacteroidia;Sphingobacteriales;Sphingobacteriaceae;Mucilaginibacter; | |
| 0 | 360 | 1 | 0 | 1 | Bacteria;Chloroflexi;KD4-96; | |
| 0 | 668 | 0 | 1 | 0 | Bacteria;Cyanobacteria;Melainabacteria;Gastranaerophilales; | |
| 0 | 0 | 41 | 33 | 0 | Bacteria;Cyanobacteria;Melainabacteria;Obscuribacterales; | |
| 0 | 1366 | 268 | 0 | 904 | Bacteria;Cyanobacteria;Oxyphotobacteria;Chloroplast; | |
| 0 | 1253 | 0 | 0 | 0 | Bacteria;Cyanobacteria;Oxyphotobacteria;Nostocales;Chroococcidiopsaceae;uncultured; | |
| 0 | 737 | 0 | 0 | 0 | Bacteria;Cyanobacteria;Oxyphotobacteria;Synechococcales;Prochlorotrichaceae;  Prochlorothrix PCC-9006; | |
| 2 | 724 | 0 | 0 | 0 | Bacteria;Epsilonbacteraeota;Campylobacteria;Campylobacterales;Campylobacteraceae;  Campylobacter; | |
| 0 | 0 | 1520 | 0 | 0 | Bacteria;Firmicutes;Bacilli;Bacillales;Bacillaceae;Anoxybacillus; | |
| 0 | 0 | 873 | 0 | 0 | Bacteria;Firmicutes;Bacilli;Bacillales;Bacillaceae;Bacillus; | |
| 0 | 0 | 239 | 0 | 0 | Bacteria;Firmicutes;Bacilli;Bacillales;Bacillaceae;Caldibacillus; | |
| 0 | 0 | 1069 | 0 | 0 | Bacteria;Firmicutes;Bacilli;Bacillales;Family X;Thermicanus; | |
| 0 | 1 | 1 | 3 | 0 | Bacteria;Firmicutes;Bacilli;Bacillales;Family XI;Gemella; | |
| 0 | 0 | 26 | 0 | 0 | Bacteria;Firmicutes;Bacilli;Bacillales;Listeriaceae;Brochothrix; | |
| 0 | 91 | 0 | 0 | 0 | Bacteria;Firmicutes;Bacilli;Bacillales;Paenibacillaceae;Paenibacillus; | |
| 2 | 845 | 1093 | 2 | 371 | Bacteria;Firmicutes;Bacilli;Bacillales;Staphylococcaceae;Staphylococcus; | |
| 0 | 31 | 9 | 1 | 0 | Bacteria;Firmicutes;Bacilli;Lactobacillales;Carnobacteriaceae;Atopostipes; | |
| 0 | 174 | 0 | 1 | 0 | Bacteria;Firmicutes;Bacilli;Lactobacillales;Carnobacteriaceae;Granulicatella; | |
| 0 | 0 | 73 | 0 | 0 | Bacteria;Firmicutes;Bacilli;Lactobacillales;Enterococcaceae;Enterococcus; | |
| 3 | 396 | 4 | 11 | 32 | Bacteria;Firmicutes;Bacilli;Lactobacillales;Streptococcaceae;Streptococcus; | |
| 0 | 6 | 9 | 3 | 69 | Bacteria;Firmicutes;Clostridia;Clostridiales;Christensenellaceae;Christensenellaceae R-7 group; | |
| 0 | 0 | 0 | 278 | 0 | Bacteria;Firmicutes;Clostridia;Clostridiales;Clostridiaceae 1;Fonticella; | |
| 0 | 0 | 5 | 0 | 0 | Bacteria;Firmicutes;Clostridia;Clostridiales;Clostridiales vadinBB60 group; | |
| 0 | 991 | 325 | 0 | 0 | Bacteria;Firmicutes;Clostridia;Clostridiales;Family XI;Anaerococcus; | |
| 184 | 384 | 0 | 0 | 0 | Bacteria;Firmicutes;Clostridia;Clostridiales;Family XI;Peptoniphilus; | |
| 1 | 0 | 0 | 0 | 0 | Bacteria;Firmicutes;Clostridia;Clostridiales;Family XIII;[Eubacterium] nodatum group; | |
| 2 | 204 | 7 | 4 | 1 | Bacteria;Firmicutes;Clostridia;Clostridiales;Lachnospiraceae;Blautia; | |
| 1 | 1 | 0 | 2 | 0 | Bacteria;Firmicutes;Clostridia;Clostridiales;Lachnospiraceae;Dorea; | |
| 0 | 197 | 3 | 0 | 0 | Bacteria;Firmicutes;Clostridia;Clostridiales;Lachnospiraceae;Fusicatenibacter; | |
| 0 | 0 | 0 | 1 | 0 | Bacteria;Firmicutes;Clostridia;Clostridiales;Lachnospiraceae;Lachnoanaerobaculum; | |
| 0 | 2 | 1 | 1 | 0 | Bacteria;Firmicutes;Clostridia;Clostridiales;Lachnospiraceae;Lachnospiraceae NK4A136 group; | |
| 0 | 0 | 0 | 0 | 1 | Bacteria;Firmicutes;Clostridia;Clostridiales;Lachnospiraceae;Lachnospiraceae UCG-001; | |
| 3 | 3 | 3 | 3 | 2 | Bacteria;Firmicutes;Clostridia;Clostridiales;Lachnospiraceae;Roseburia; | |
| 0 | 687 | 2 | 2 | 1 | Bacteria;Firmicutes;Clostridia;Clostridiales;Lachnospiraceae;[Eubacterium] hallii group; | |
| 0 | 1 | 0 | 0 | 1 | Bacteria;Firmicutes;Clostridia;Clostridiales;Lachnospiraceae;[Ruminococcus] gnavus group; | |
| 0 | 0 | 5 | 0 | 1 | Bacteria;Firmicutes;Clostridia;Clostridiales;Lachnospiraceae;uncultured; | |
| 0 | 0 | 0 | 0 | 1 | Bacteria;Firmicutes;Clostridia;Clostridiales;Peptostreptococcaceae;Intestinibacter; | |
| 0 | 0 | 0 | 0 | 1 | Bacteria;Firmicutes;Clostridia;Clostridiales;Peptostreptococcaceae;Peptostreptococcus; | |
| 5 | 5 | 13 | 14 | 7 | Bacteria;Firmicutes;Clostridia;Clostridiales;Ruminococcaceae;Faecalibacterium; | |
| 1 | 21 | 204 | 1 | 0 | Bacteria;Firmicutes;Clostridia;Clostridiales;Ruminococcaceae;Oscillibacter; | |
| 0 | 0 | 4 | 0 | 0 | Bacteria;Firmicutes;Clostridia;Clostridiales;Ruminococcaceae;Ruminococcaceae NK4A214 group; | |
| 1 | 1 | 2 | 1 | 1 | Bacteria;Firmicutes;Clostridia;Clostridiales;Ruminococcaceae;Ruminococcaceae UCG-005; | |
| 0 | 0 | 4 | 8 | 0 | Bacteria;Firmicutes;Clostridia;Clostridiales;Ruminococcaceae;Ruminococcaceae UCG-014; | |
| 0 | 6 | 3 | 1 | 0 | Bacteria;Firmicutes;Clostridia;Clostridiales;Ruminococcaceae;Subdoligranulum; | |
| 0 | 6 | 13 | 4 | 1 | Bacteria;Firmicutes;Clostridia;Clostridiales;Ruminococcaceae;  [Eubacterium] coprostanoligenes group; | |
| 0 | 0 | 1 | 1 | 179 | Bacteria;Firmicutes;Clostridia;Clostridiales;Ruminococcaceae;uncultured; | |
| 0 | 0 | 88 | 0 | 0 | Bacteria;Firmicutes;Clostridia;Thermoanaerobacterales;Family III;Thermoanaerobacterium; | |
| 1 | 0 | 0 | 0 | 1 | Bacteria;Firmicutes;Negativicutes;Selenomonadales;Veillonellaceae;Anaeroglobus; | |
| 1 | 1 | 6 | 0 | 0 | Bacteria;Firmicutes;Negativicutes;Selenomonadales;Veillonellaceae;Megasphaera; | |
| 4 | 1 | 0 | 0 | 0 | Bacteria;Firmicutes;Negativicutes;Selenomonadales;Veillonellaceae;Selenomonas 3; | |
| 4 | 36 | 3 | 492 | 184 | Bacteria;Firmicutes;Negativicutes;Selenomonadales;Veillonellaceae;Veillonella; | |
| 0 | 0 | 0 | 2 | 0 | Bacteria;Fusobacteria;Fusobacteriia;Fusobacteriales;Fusobacteriaceae;Fusobacterium; | |
| 1 | 0 | 0 | 1 | 0 | Bacteria;Fusobacteria;Fusobacteriia;Fusobacteriales;Leptotrichiaceae;Leptotrichia; | |
| 0 | 0 | 0 | 4 | 0 | Bacteria;Patescibacteria;Saccharimonadia;Saccharimonadales; | |
| 0 | 0 | 0 | 1 | 0 | Bacteria;Patescibacteria;Saccharimonadia;Saccharimonadales;Saccharimonadaceae; | |
| 0 | 0 | 0 | 0 | 13 | Bacteria;Proteobacteria;Alphaproteobacteria;Acetobacterales;Acetobacteraceae;Acidisoma; | |
| 0 | 0 | 134 | 0 | 0 | Bacteria;Proteobacteria;Alphaproteobacteria;Acetobacterales;Acetobacteraceae;uncultured; | |
| 0 | 2 | 0 | 0 | 0 | Bacteria;Proteobacteria;Alphaproteobacteria;Caulobacterales;Caulobacteraceae;Brevundimonas; | |
| 0 | 0 | 28 | 0 | 0 | Bacteria;Proteobacteria;Alphaproteobacteria;Caulobacterales;Caulobacteraceae;Caulobacter; | |
| 0 | 2 | 0 | 0 | 0 | Bacteria;Proteobacteria;Alphaproteobacteria;Rhizobiales;Beijerinckiaceae;Bosea; | |
| 1 | 0 | 0 | 0 | 0 | Bacteria;Proteobacteria;Alphaproteobacteria;Rhizobiales;Beijerinckiaceae;Methylobacterium; | |
| 213 | 44 | 426 | 0 | 0 | Bacteria;Proteobacteria;Alphaproteobacteria;Rhizobiales;Rhizobiaceae;  Allorhizobium-Neorhizobium-Pararhizobium-Rhizobium; | |
| 305 | 158 | 468 | 532 | 864 | Bacteria;Proteobacteria;Alphaproteobacteria;Rhizobiales;Rhizobiaceae;Brucella; | |
| 0 | 2 | 0 | 1 | 0 | Bacteria;Proteobacteria;Alphaproteobacteria;Rhizobiales;Rhizobiaceae;Mesorhizobium; | |
| 804 | 1137 | 54 | 119 | 440 | Bacteria;Proteobacteria;Alphaproteobacteria;Rhizobiales;Rhizobiaceae;Ochrobactrum; | |
| 1 | 1 | 0 | 0 | 0 | Bacteria;Proteobacteria;Alphaproteobacteria;Rhizobiales;Rhizobiaceae;Phyllobacterium; | |
| 0 | 1 | 0 | 0 | 0 | Bacteria;Proteobacteria;Alphaproteobacteria;Rhizobiales;Xanthobacteraceae;Bradyrhizobium; | |
| 0 | 147 | 0 | 0 | 0 | Bacteria;Proteobacteria;Alphaproteobacteria;Sphingomonadales;Sphingomonadaceae;  Novosphingobium; | |
| 0 | 0 | 1 | 0 | 0 | Bacteria;Proteobacteria;Alphaproteobacteria;Sphingomonadales;Sphingomonadaceae;  Sphingobium; | |
| 74 | 576 | 0 | 51 | 18 | Bacteria;Proteobacteria;Alphaproteobacteria;Sphingomonadales;Sphingomonadaceae  ;Sphingomonas; | |
| 0 | 0 | 0 | 124 | 0 | Bacteria;Proteobacteria;Deltaproteobacteria;Myxococcales;mle1-27; | |
| 0 | 0 | 135 | 0 | 0 | Bacteria;Proteobacteria;Gammaproteobacteria;Betaproteobacteriales;Burkholderiaceae;  Burkholderia-Caballeronia-Paraburkholderia; | |
| 0 | 122 | 0 | 0 | 228 | Bacteria;Proteobacteria;Gammaproteobacteria;Betaproteobacteriales;Burkholderiaceae;  Hydrogenophaga; | |
| 0 | 445 | 515 | 1 | 0 | Bacteria;Proteobacteria;Gammaproteobacteria;Betaproteobacteriales;Burkholderiaceae;Massilia; | |
| 0 | 1 | 1 | 0 | 0 | Bacteria;Proteobacteria;Gammaproteobacteria;Betaproteobacteriales;Burkholderiaceae;  Parasutterella; | |
| 0 | 44 | 50 | 0 | 0 | Bacteria;Proteobacteria;Gammaproteobacteria;Betaproteobacteriales;Burkholderiaceae;  Schlegelella; | |
| 0 | 25 | 168 | 0 | 0 | Bacteria;Proteobacteria;Gammaproteobacteria;Betaproteobacteriales;Burkholderiaceae;  Tepidimonas; | |
| 0 | 11 | 0 | 0 | 0 | Bacteria;Proteobacteria;Gammaproteobacteria;Betaproteobacteriales;Burkholderiaceae;uncultured; | |
| 0 | 36 | 0 | 0 | 0 | Bacteria;Proteobacteria;Gammaproteobacteria;Betaproteobacteriales;Hydrogenophilaceae;  Hydrogenophilus; | |
| 0 | 0 | 127 | 0 | 0 | Bacteria;Proteobacteria;Gammaproteobacteria;Betaproteobacteriales;Hydrogenophilaceae;  Tepidiphilus; | |
| 2 | 0 | 0 | 8 | 0 | Bacteria;Proteobacteria;Gammaproteobacteria;Betaproteobacteriales;Neisseriaceae;Neisseria; | |
| 0 | 307 | 0 | 0 | 49 | Bacteria;Proteobacteria;Gammaproteobacteria;Betaproteobacteriales;Neisseriaceae;uncultured; | |
| 0 | 0 | 491 | 0 | 0 | Bacteria;Proteobacteria;Gammaproteobacteria;Betaproteobacteriales;Nitrosomonadaceae;  Ellin6067; | |
| 0 | 838 | 0 | 0 | 0 | Bacteria;Proteobacteria;Gammaproteobacteria;CCD24; | |
| 0 | 0 | 0 | 9 | 3 | Bacteria;Proteobacteria;Gammaproteobacteria;Enterobacteriales;Enterobacteriaceae;Enterobacter; | |
| 0 | 0 | 9 | 7 | 69 | Bacteria;Proteobacteria;Gammaproteobacteria;Enterobacteriales;Enterobacteriaceae;  Escherichia-Shigella; | |
| 0 | 0 | 0 | 0 | 1 | Bacteria;Proteobacteria;Gammaproteobacteria;Gammaproteobacteria Incertae Sedis;  Unknown Family;Acidibacter; | |
| 0 | 454 | 0 | 0 | 0 | Bacteria;Proteobacteria;Gammaproteobacteria;Oceanospirillales;Alcanivoracaceae;Alcanivorax; | |
| 0 | 1 | 0 | 0 | 0 | Bacteria;Proteobacteria;Gammaproteobacteria;Pasteurellales;Pasteurellaceae;Actinobacillus; | |
| 1 | 2 | 0 | 4 | 0 | Bacteria;Proteobacteria;Gammaproteobacteria;Pasteurellales;Pasteurellaceae;Haemophilus; | |
| 7953 | 9411 | 5126 | 3973 | 4327 | Bacteria;Proteobacteria;Gammaproteobacteria;Pseudomonadales;Moraxellaceae;Acinetobacter; | |
| 0 | 665 | 483 | 1 | 423 | Bacteria;Proteobacteria;Gammaproteobacteria;Pseudomonadales;Moraxellaceae;Enhydrobacter; | |
| 0 | 0 | 30 | 0 | 0 | Bacteria;Proteobacteria;Gammaproteobacteria;Pseudomonadales;Moraxellaceae;Moraxella; | |
| 1 | 3 | 507 | 375 | 2 | Bacteria;Proteobacteria;Gammaproteobacteria;Pseudomonadales;Pseudomonadaceae;  Pseudomonas; | |
| 0 | 0 | 0 | 1 | 0 | Bacteria;Proteobacteria;Gammaproteobacteria;Salinisphaerales;Solimonadaceae;Polycyclovorans; | |
| 0 | 0 | 0 | 1 | 0 | Bacteria;Proteobacteria;Gammaproteobacteria;Xanthomonadales;Rhodanobacteraceae;Dokdonella; | |
| 0 | 0 | 1 | 0 | 0 | Bacteria;Proteobacteria;Gammaproteobacteria;Xanthomonadales;Rhodanobacteraceae;uncultured; | |
| 0 | 0 | 115 | 0 | 0 | Bacteria;Proteobacteria;Gammaproteobacteria;Xanthomonadales;Xanthomonadaceae;  Pseudoxanthomonas; | |
| 641 | 53 | 29 | 15689 | 17 | Bacteria;Proteobacteria;Gammaproteobacteria;Xanthomonadales;Xanthomonadaceae;  Stenotrophomonas; | |
| 0 | 0 | 0 | 1 | 0 | Bacteria;Verrucomicrobia;Verrucomicrobiae;Pedosphaerales;Pedosphaeraceae; | |
| 0 | 1 | 0 | 0 | 0 | No Relative | |

**Supplemental Table 5.** Contaminant taxa associated with the indicated extraction batches inferred using the R package decontam ([27](#_ENREF_27)). We used a probability threshold of 0.5 to accept the alternate hypothesis (the OTU is a contaminant). For simplification, only those OTUs identified as potential extraction-related contaminants are shown. OTU, Operational Taxonomic Unit; CF, cystic fibrosis; COPD, chronic obstructive pulmonary disease; PEG, polyethylene glycol-based method.

| **CF-PEG** |  |  |
| --- | --- | --- |
| **OTU** | **Fisher’s combined probability** | **Contaminant** |
| Corynebacterium 1 | 0.0347355257326935 | TRUE |
| Lawsonella | 0.269041760209023 | TRUE |
| Arthrobacter | 0.189479424703154 | TRUE |
| KocuriaS | 0.0564055113751904 | TRUE |
| Muribaculaceae | 0.29631803316677 | TRUE |
| Prevotella 6 | 0.485668753322839 | TRUE |
| Prevotella | 0.0860466399360806 | TRUE |
| KD4-96 | 0.105940696781658 | TRUE |
| Lactobacillus | 0.36366403943348 | TRUE |
| Ruminococcaceae UCG-005 | 0.36461178494507 | TRUE |
| Megasphaera | 0.201214466015468 | TRUE |
| uncultured_Acetobacteraceae | 0.0792427399689728 | TRUE |
| Allorhizobium-Neorhizobium-Pararhizobium-Rhizobium | 0.0696973183417979 | TRUE |
| Brucella | 0.0469832825796346 | TRUE |
| Ochrobactrum | 0.438456808819252 | TRUE |
| Sphingomonas | 0.405563752977576 | TRUE |
| Acidovorax | 0.491720931546671 | TRUE |
| Massilia | 0.326811154964623 | TRUE |
| Schlegelella | 0.360653615536259 | TRUE |
| Neisseria | 0.439277259686118 | TRUE |
| uncultured_Neisseriaceae | 0.344951626558937 | TRUE |
| SC-I-84 | 0.379966871457755 | TRUE |
| Serratia | 0.361106277228807 | TRUE |
| Acinetobacter | 0.0475632269288317 | TRUE |

| **CF-QIAamp microbiome kit & host depletion** |  |  |  |
| --- | --- | --- | --- |
| **OTU** | **Fisher’s combined probability** | **Contaminant** | |
| Corynebacterium | 0.425469848446393 | TRUE |  |
| Brevibacterium | 0.335715066735371 | TRUE |  |
| Microbacterium | 0.345265104468305 | TRUE |  |
| Rothia | 0.349446939097703 | TRUE |  |
| Proteiniphilum | 0.467655858780375 | TRUE |  |
| Prevotella 7 | 0.43377667515819 | TRUE |  |
| Chryseobacterium | 0.43183240539927 | TRUE |  |
| Elizabethkingia | 0.348332096619044 | TRUE |  |
| Campylobacter | 0.348736611880512 | TRUE |  |
| Gemella | 0.426224714420516 | TRUE |  |
| Paenibacillus | 0.358289712412125 | TRUE |  |
| Atopostipes | 0.359184309904842 | TRUE |  |
| Clostridiales vadinBB60 group | 0.479155230978555 | TRUE |  |
| Peptoniphilus | 0.322370596156783 | TRUE |  |
| Dorea | 0.422439815188717 | TRUE |  |
| Fusicatenibacter | 0.406662617296962 | TRUE |  |
| Lachnospiraceae NK4A136 group | 0.42342093452335 | TRUE |  |
| Roseburia | 0.441970960337543 | TRUE |  |
| [Ruminococcus] gnavus group | 0.379095128220932 | TRUE |  |
| Ruminococcaceae UCG-005 | 0.429543277439136 | TRUE |  |
| Subdoligranulum | 0.439496057331912 | TRUE |  |
| Selenomonas 3 | 0.357638468528928 | TRUE |  |
| Bosea | 0.323475257264182 | TRUE |  |
| Allorhizobium-Neorhizobium-Pararhizobium-Rhizobium | 0.485689416587769 | TRUE |  |
| Schlegelella | 0.325512516640664 | TRUE |  |
| Hydrogenophilus | 0.448250460993046 | TRUE |  |
| Actinobacillus | 0.308296223719893 | TRUE |  |
| No Relative | 0.378075664249647 | TRUE |  |

| **CF-QIAamp microbiome kit** |  |  | |
| --- | --- | --- | --- |
| **OTU** | **Fisher’s combined probability** | **Contaminant** | |
| Bacillus | 0.397155384579083 | TRUE |  |
| Gemella | 0.433258202472804 | TRUE |  |
| Fusicatenibacter | 0.441042370740697 | TRUE |  |
| [Eubacterium] hallii group | 0.494968946319866 | TRUE |  |
| Oscillibacter | 0.441042370740697 | TRUE |  |
| Ruminococcaceae UCG-005 | 0.436401821318659 | TRUE |  |
| Megasphaera | 0.459080402266993 | TRUE |  |
| Allorhizobium-Neorhizobium-Pararhizobium-Rhizobium | 0.437082794572097 | TRUE |  |
| Diaphorobacter | 0.451652958248916 | TRUE |  |
| uncultured_Rhodanobacteraceae | 0.44193961431826 | TRUE |  |

| **COPD-PEG** |  |  |
| --- | --- | --- |
| **OTU** | **Fisher’s combined probability** | **Contaminant** |
| Lawsonella | 0.392604864902295 | TRUE |
| Porphyromonas | 0.396696552550234 | TRUE |
| Prevotella 7 | 0.272952002833224 | TRUE |
| uncultured_Saprospiraceae | 0.396539859810367 | TRUE |
| Chryseobacterium | 0.39629426784003 | TRUE |
| Ruminococcaceae UCG-005 | 0.29240604148044 | TRUE |
| Ruminococcaceae UCG-014 | 0.386775936367603 | TRUE |
| Solobacterium | 0.351412547743255 | TRUE |
| Veillonella | 0.434568773750999 | TRUE |
| Saccharimonadales | 0.477563974021997 | TRUE |
| Saccharimonadaceae | 0.478068833032085 | TRUE |
| Caulobacter | 0.18271237270159 | TRUE |
| Neisseria | 0.477150459853679 | TRUE |
| Enterobacter | 0.289856874603144 | TRUE |
| Escherichia-Shigella | 0.4372020010683 | TRUE |

| **COPD-QIAamp microbiome kit** |  |  |
| --- | --- | --- |
| **OTU** | **Fisher’s combined probability** | **Contaminant** |
| Rothia | 0.403368926139667 | TRUE |
| Muribaculaceae | 0.398873616367976 | TRUE |
| Christensenellaceae R-7 group | 0.431763692155445 | TRUE |
| Lachnospiraceae UCG-001 | 0.334865155151772 | TRUE |
| Roseburia | 0.389144255529944 | TRUE |
| Intestinibacter | 0.405591137935601 | TRUE |
| Oscillibacter | 0.393520318152498 | TRUE |
| Ruminococcaceae UCG-005 | 0.400953596535595 | TRUE |
| [Eubacterium] coprostanoligenes group | 0.47656166760472 | TRUE |
| uncultured_Ruminococcaceae | 0.434214945689291 | TRUE |
| Anaeroglobus | 0.364987005649521 | TRUE |
| Selenomonas 4 | 0.216231392359354 | TRUE |
| Veillonella | 0.474767537917088 | TRUE |
| Acidisoma | 0.410361061409205 | TRUE |
| Enterobacter | 0.410138157087779 | TRUE |
| Escherichia-Shigella | 0.353776545055763 | TRUE |
|  |  |  |

**Supplemental Table 6.** Alpha (richness) diversity observed in the different samples including in this study. CF, cystic fibrosis; HS, healthy smoker; COPD, chronic obstructive pulmonary disease; NEC, negative extraction control; PEG, polyethylene glycol; Q, QIAamp microbiome kit; QH, QIAamp microbiome kit with DNA host depletion.

| Sample ID | Sample group | Extraction method | Richness (number of OTUs) |
| --- | --- | --- | --- |
| P52 | CF patient | PEG | 50 |
| P36 | CF patient | PEG | 47 |
| P39 | CF patient | PEG | 40 |
| P43 | CF patient | PEG | 57 |
| P47 | CF patient | PEG | 38 |
| P58 | CF patient | PEG | 24 |
| PCNT | NEC | PEG | 30 |
| QH52 | CF patient | QH | 53 |
| QH36 | CF patient | QH | 63 |
| QH39 | CF patient | QH | 63 |
| QH43 | CF patient | QH | 44 |
| QH47 | CF patient | QH | 61 |
| QH58 | CF patient | QH | 54 |
| QHCNT | NEC | QH | 71 |
| QO39 | CF patient | Q | 80 |
| QO43 | CF patient | Q | 87 |
| QO47 | CF patient | Q | 75 |
| QO58 | CF patient | Q | 68 |
| QCNT | NEC | Q | 60 |
| 11850 | HS | PEG | 70 |
| 18177 | COPD-GOLD1 | PEG | 79 |
| 12306 | COPD-GOLD2 | PEG | 88 |
| 11026 | COPD-GOLD3 | PEG | 76 |
| 19677 | COPD-GOLD4 | PEG | 69 |
| CNT168 | NEC | PEG | 49 |
| Q11850 | HS | Q | 62 |
| Q18177 | COPD-GOLD1 | Q | 65 |
| Q12306 | COPD-GOLD2 | Q | 88 |
| Q11026 | COPD-GOLD3 | Q | 73 |
| Q19677 | COPD-GOLD4 | Q | 64 |
| CNT278 | NEC | Q | 45 |

**Supplemental Table 7.** Correlation between the clinical microbiology findings and the 16S profiles from the PEG-associated DNA extracts. MOF, mixed oral flora; *E. coli*, *Escherichia coli*; *S. aureus*, *Staphylococcus aureus*; *E. Sp*, *Enterobacter sp*; *K. sp*, *Klebsiella sp.* * Bacterial DNA concentration in sample P58 was similar to that of the corresponding negative extraction control. As mixed oral flora we considered the number of reads of the OTUs associated with the *Streptococcus*, *Lactobacillus* and *Prevotella* taxa.

| Sample ID | Sample group | Clinical microbiology | 16S profiles PEG method (reads) |
| --- | --- | --- | --- |
| P52 | CF patient | MOF | MOF (102) |
| P36 | CF patient | *E.coli*, MOF, *S. aureus* | *Escherichia-Shigella* (1,718), MOF(15,021), *Staphylococcus* (285) |
| P39 | CF patient | MOF | MOF(1,194) |
| P58* | CF patient | *E. sp*, *K. Sp*, MOF | *Enterobacter* (0), *Klebsiella* (0), MOF(43) |
| 11026 | COPD-GOLD3 | *Pseudomonas aeruginosa* | *Pseudomonas* (1,259) |

**Supplemental Table 8.** Technical information related to the TaqMan® assays and provided following the MIQE guidelines([28](#_ENREF_28)). Standard curves were generated with genomic DNA extracts from *Pseudomonas aeruginosa* (total bacteria TaqMan® assay) or *Stenotrophomonas maltophilia* (*Stenotrophomonas maltophilia* TaqMan® assay) as indicated in the material and methods section of this manuscript. 16S copy number was estimated using the following data values. For total bacteria DNA, we considered a median total length of *Pseudomonas aeruginosa* genome of 6.60808 Mb (<https://www.ncbi.nlm.nih.gov/genome/?term=Pseudomonas%20aeruginosa%5BOrganism%5D&cmd=DetailsSearch>), the average molecular mass of one base pair of 660 g/mol and the number of rRNA operons of 4 ([29](#_ENREF_29)). For the *Stenotrophomonas maltophilia* specific assay, we considered a median total length of its genome of 4.62588 Mb ([https://www.ncbi.nlm.nih.gov/genome/?term=Stenotrophomonas%20maltophilia[Organism]&cmd=DetailsSearch](https://www.ncbi.nlm.nih.gov/genome/?term=Stenotrophomonas%20maltophilia%5bOrganism%5d&cmd=DetailsSearch)) and the presence of 4 copies of the rRNA operon per genome ([30](#_ENREF_30)).

| Total bacteria |  |
| --- | --- |
| Oligonucleotides | Forward *5’-AATAAGCACCGGCTAACTTCGT* |
|  | Reverse *5’-AACGCTTGCACCCTTCGTAT* |
|  | Probe 5’-FAM-*CCAGCAGCCGCGGT*-MGB |
| Amplicon length | 58 bp |
| Limit of detection | 3 pg (1,657 16S copies) *Pseudomonas aeruginosa* genomic DNA |
| Repeatability (intra assay variation) | Coefficient of variation (CV) <1% |
| Reproducibility (inter assay variation) | CV 2-3% |
| Calibration curve | Slope: -3.223 |
|  | Y-intercept: 30.87  PCR efficiency: 104.323  R^2^ 0.997  Linear dynamic range (16S copies): 5 pg (2,762)-2ng (1,104,800) of *Pseudomonas aeruginosa* genomic DNA |

| *Stenotrophomonas maltophilia* |  |
| --- | --- |
| Oligonucleotides | Forward *5’-CATCAGGCTGACGTGGAACTT* |
|  | Reverse *5’-CCAAGCGCACGCAGAAG* |
|  | Probe 5’-FAM-*ACGACATCTGGTTGGC*-MGB |
| Amplicon length | 80 bp |
| Limit of detection | <0.1 pg (<79 16S copies) *Stenotrophomonas maltophilia* genomic DNA |
| Repeatability (intra assay variation) | CV <1% |
| Reproducibility (inter assay variation) | CV <1% |
| Calibration curve | Slope: -3.542 |
|  | Y-intercept: 43.6  PCR efficiency: 91.58  R^2^ 0.999  Linear dynamic range (16S copies): 0.1 pg (79) - 200pg (157,461) of *Stenotrophomonas maltophilia* genomic DNA |

**References**

1. Marchesi JR, Sato T, Weightman AJ, Martin TA, Fry JC, Hiom SJ, et al. Design and evaluation of useful bacterium-specific PCR primers that amplify genes coding for bacterial 16S rRNA. *Applied and environmental microbiology* (1998) 64(2):795-9. PubMed PMID: 9464425; PubMed Central PMCID: PMC106123.

2. Zemanick ET, Wagner BD, Robertson CE, Ahrens RC, Chmiel JF, Clancy JP, et al. Airway microbiota across age and disease spectrum in cystic fibrosis. *The European respiratory journal* (2017) 50(5). doi: 10.1183/13993003.00832-2017. PubMed PMID: 29146601; PubMed Central PMCID: PMC5935257.

3. Pattaroni C, Watzenboeck ML, Schneidegger S, Kieser S, Wong NC, Bernasconi E, et al. Early-Life Formation of the Microbial and Immunological Environment of the Human Airways. *Cell host & microbe* (2018) 24(6):857-65 e4. doi: 10.1016/j.chom.2018.10.019. PubMed PMID: 30503510.

4. Willner D, Daly J, Whiley D, Grimwood K, Wainwright CE, Hugenholtz P. Comparison of DNA extraction methods for microbial community profiling with an application to pediatric bronchoalveolar lavage samples. *PLoS One* (2012) 7(4):e34605. doi: 10.1371/journal.pone.0034605. PubMed PMID: 22514642; PubMed Central PMCID: PMC3326054.

5. Marsh RL, Kaestli M, Chang AB, Binks MJ, Pope CE, Hoffman LR, et al. The microbiota in bronchoalveolar lavage from young children with chronic lung disease includes taxa present in both the oropharynx and nasopharynx. *Microbiome* (2016) 4(1):37. doi: 10.1186/s40168-016-0182-1. PubMed PMID: 27388563; PubMed Central PMCID: PMC4936249.

6. Laguna TA, Wagner BD, Williams CB, Stevens MJ, Robertson CE, Welchlin CW, et al. Airway Microbiota in Bronchoalveolar Lavage Fluid from Clinically Well Infants with Cystic Fibrosis. *PLoS One* (2016) 11(12):e0167649. doi: 10.1371/journal.pone.0167649. PubMed PMID: 27930727; PubMed Central PMCID: PMC5145204 receives grant funding from the Cystic Fibrosis Foundation. (2) E.T.Z./B.D.W./J.K.H. - receive grant funding from the Cystic Fibrosis Foundation. Otherwise, the authors have declared that no other competing interests exist. In addition, this does not alter our adherence to PLOS ONE policies on sharing data and materials.

7. Dickson RP, Singer BH, Newstead MW, Falkowski NR, Erb-Downward JR, Standiford TJ, et al. Enrichment of the lung microbiome with gut bacteria in sepsis and the acute respiratory distress syndrome. *Nature microbiology* (2016) 1(10):16113. doi: 10.1038/nmicrobiol.2016.113. PubMed PMID: 27670109; PubMed Central PMCID: PMC5076472.

8. Han MK, Zhou Y, Murray S, Tayob N, Noth I, Lama VN, et al. Lung microbiome and disease progression in idiopathic pulmonary fibrosis: an analysis of the COMET study. *The Lancet Respiratory medicine* (2014) 2(7):548-56. doi: 10.1016/S2213-2600(14)70069-4. PubMed PMID: 24767767; PubMed Central PMCID: PMC4142525.

9. Esther CR, Jr., Muhlebach MS, Ehre C, Hill DB, Wolfgang MC, Kesimer M, et al. Mucus accumulation in the lungs precedes structural changes and infection in children with cystic fibrosis. *Science translational medicine* (2019) 11(486). doi: 10.1126/scitranslmed.aav3488. PubMed PMID: 30944166; PubMed Central PMCID: PMC6566903.

10. Erb-Downward JR, Thompson DL, Han MK, Freeman CM, McCloskey L, Schmidt LA, et al. Analysis of the lung microbiome in the "healthy" smoker and in COPD. *PLoS One* (2011) 6(2):e16384. doi: 10.1371/journal.pone.0016384. PubMed PMID: 21364979; PubMed Central PMCID: PMC3043049.

11. Bernasconi E, Pattaroni C, Koutsokera A, Pison C, Kessler R, Benden C, et al. Airway Microbiota Determines Innate Cell Inflammatory or Tissue Remodeling Profiles in Lung Transplantation. *American journal of respiratory and critical care medicine* (2016) 194(10):1252-63. doi: 10.1164/rccm.201512-2424OC. PubMed PMID: 27248293.

12. Frayman KB, Armstrong DS, Carzino R, Ferkol TW, Grimwood K, Storch GA, et al. The lower airway microbiota in early cystic fibrosis lung disease: a longitudinal analysis. *Thorax* (2017) 72(12):1104-12. doi: 10.1136/thoraxjnl-2016-209279. PubMed PMID: 28280235.

13. Kloepfer KM, Deschamp AR, Ross SE, Peterson-Carmichael SL, Hemmerich CM, Rusch DB, et al. In children, the microbiota of the nasopharynx and bronchoalveolar lavage fluid are both similar and different. *Pediatric pulmonology* (2018) 53(4):475-82. doi: 10.1002/ppul.23953. PubMed PMID: 29405661; PubMed Central PMCID: PMC6542268.

14. Jorth P, Ehsan Z, Rezayat A, Caldwell E, Pope C, Brewington JJ, et al. Direct Lung Sampling Indicates That Established Pathogens Dominate Early Infections in Children with Cystic Fibrosis. *Cell reports* (2019) 27(4):1190-204 e3. doi: 10.1016/j.celrep.2019.03.086. PubMed PMID: 31018133; PubMed Central PMCID: PMC6668708.

15. Pragman AA, Kim HB, Reilly CS, Wendt C, Isaacson RE. The lung microbiome in moderate and severe chronic obstructive pulmonary disease. *PLoS One* (2012) 7(10):e47305. doi: 10.1371/journal.pone.0047305. PubMed PMID: 23071781; PubMed Central PMCID: PMC3469539.

16. Ahmed B, Cox MJ, Cuthbertson L, James PL, Cookson WOC, Davies JC, et al. Comparison of the upper and lower airway microbiota in children with chronic lung diseases. *PLoS One* (2018) 13(8):e0201156. doi: 10.1371/journal.pone.0201156. PubMed PMID: 30071000; PubMed Central PMCID: PMC6071972.

17. May AK, Brady JS, Romano-Keeler J, Drake WP, Norris PR, Jenkins JM, et al. A pilot study of the noninvasive assessment of the lung microbiota as a potential tool for the early diagnosis of ventilator-associated pneumonia. *Chest* (2015) 147(6):1494-502. doi: 10.1378/chest.14-1687. PubMed PMID: 25474571; PubMed Central PMCID: PMC4451706.

18. Schneeberger PHH, Prescod J, Levy L, Hwang D, Martinu T, Coburn B. Microbiota analysis optimization for human bronchoalveolar lavage fluid. *Microbiome* (2019) 7(1):141. doi: 10.1186/s40168-019-0755-x. PubMed PMID: 31665066; PubMed Central PMCID: PMC6821041.

19. Wang H, Zhou Q, Dai W, Feng X, Lu Z, Yang Z, et al. Lung Microbiota and Pulmonary Inflammatory Cytokines Expression Vary in Children With Tracheomalacia and Adenoviral or Mycoplasma pneumoniae Pneumonia. *Frontiers in pediatrics* (2019) 7:265. doi: 10.3389/fped.2019.00265. PubMed PMID: 31316955; PubMed Central PMCID: PMC6611399.

20. Kyo M, Nishioka K, Nakaya T, Kida Y, Tanabe Y, Ohshimo S, et al. Unique patterns of lower respiratory tract microbiota are associated with inflammation and hospital mortality in acute respiratory distress syndrome. *Respiratory research* (2019) 20(1):246. doi: 10.1186/s12931-019-1203-y. PubMed PMID: 31694652; PubMed Central PMCID: PMC6836399.

21. Wen Y, Xiao F, Wang C, Wang Z. The impact of different methods of DNA extraction on microbial community measures of BALF samples based on metagenomic data. *American journal of translational research* (2016) 8(3):1412-25. PubMed PMID: 27186268; PubMed Central PMCID: PMC4858570.

22. Charlson ES, Bittinger K, Haas AR, Fitzgerald AS, Frank I, Yadav A, et al. Topographical continuity of bacterial populations in the healthy human respiratory tract. *American journal of respiratory and critical care medicine* (2011) 184(8):957-63. doi: 10.1164/rccm.201104-0655OC. PubMed PMID: 21680950; PubMed Central PMCID: PMC3208663.

23. Gomes S, Cavadas B, Ferreira JC, Marques PI, Monteiro C, Sucena M, et al. Profiling of lung microbiota discloses differences in adenocarcinoma and squamous cell carcinoma. *Scientific reports* (2019) 9(1):12838. doi: 10.1038/s41598-019-49195-w. PubMed PMID: 31492894; PubMed Central PMCID: PMC6731246.

24. Borewicz K, Pragman AA, Kim HB, Hertz M, Wendt C, Isaacson RE. Longitudinal analysis of the lung microbiome in lung transplantation. *FEMS microbiology letters* (2013) 339(1):57-65. doi: 10.1111/1574-6968.12053. PubMed PMID: 23173619; PubMed Central PMCID: PMC3546157.

25. Tong X, Su F, Xu X, Xu H, Yang T, Xu Q, et al. Alterations to the Lung Microbiome in Idiopathic Pulmonary Fibrosis Patients. *Frontiers in cellular and infection microbiology* (2019) 9:149. doi: 10.3389/fcimb.2019.00149. PubMed PMID: 31165050; PubMed Central PMCID: PMC6536613.

26. Renwick J, McNally P, John B, DeSantis T, Linnane B, Murphy P, et al. The microbial community of the cystic fibrosis airway is disrupted in early life. *PLoS One* (2014) 9(12):e109798. doi: 10.1371/journal.pone.0109798. PubMed PMID: 25526264; PubMed Central PMCID: PMC4272276.

27. Davis NM, Proctor DM, Holmes SP, Relman DA, Callahan BJ. Simple statistical identification and removal of contaminant sequences in marker-gene and metagenomics data. *Microbiome* (2018) 6(1):226. doi: 10.1186/s40168-018-0605-2. PubMed PMID: 30558668; PubMed Central PMCID: PMC6298009.

28. Bustin SA, Benes V, Garson JA, Hellemans J, Huggett J, Kubista M, et al. The MIQE guidelines: minimum information for publication of quantitative real-time PCR experiments. *Clinical chemistry* (2009) 55(4):611-22. doi: 10.1373/clinchem.2008.112797. PubMed PMID: 19246619.

29. Bodilis J, Nsigue-Meilo S, Besaury L, Quillet L. Variable copy number, intra-genomic heterogeneities and lateral transfers of the 16S rRNA gene in Pseudomonas. *PLoS One* (2012) 7(4):e35647. doi: 10.1371/journal.pone.0035647. PubMed PMID: 22545126; PubMed Central PMCID: PMC3335818.

30. Crossman LC, Gould VC, Dow JM, Vernikos GS, Okazaki A, Sebaihia M, et al. The complete genome, comparative and functional analysis of Stenotrophomonas maltophilia reveals an organism heavily shielded by drug resistance determinants. *Genome biology* (2008) 9(4):R74. doi: 10.1186/gb-2008-9-4-r74. PubMed PMID: 18419807; PubMed Central PMCID: PMC2643945.
